# An immuno-epidemiological model with waning immunity after infection or vaccination

**DOI:** 10.1007/s00285-024-02090-z

**Published:** 2024-04-26

**Authors:** Georgi Angelov, Raimund Kovacevic, Nikolaos I. Stilianakis, Vladimir M. Veliov

**Affiliations:** 1 Institute of Statistics and Mathematical Methods in Economics, Vienna University of Technology, Vienna, Austria; 2 Department for Economy and Health, University for Continuing Education Krems, Krems an der Donau, Austria; 3 European Commission, Joint Research Centre (JRC), Ispra, Italy; 4 Department of Biometry and Epidemiology, University of Erlangen-Nuremberg, Erlangen, Germany

**Keywords:** 92D30, 93C95, 93C20, 92-10

## Abstract

In epidemics, waning immunity is common after infection or vaccination of individuals. Immunity levels are highly heterogeneous and dynamic. This work presents an immuno-epidemiological model that captures the fundamental dynamic features of immunity acquisition and wane after infection or vaccination and analyzes mathematically its dynamical properties. The model consists of a system of first order partial differential equations, involving nonlinear integral terms and different transfer velocities. Structurally, the equation may be interpreted as a Fokker-Planck equation for a piecewise deterministic process. However, unlike the usual models, our equation involves nonlocal effects, representing the infectivity of the whole environment. This, together with the presence of different transfer velocities, makes the proved existence of a solution novel and nontrivial. In addition, the asymptotic behavior of the model is analyzed based on the obtained qualitative properties of the solution. An optimal control problem with objective function including the total number of deaths and costs of vaccination is explored. Numerical results describe the dynamic relationship between contact rates and optimal solutions. The approach can contribute to the understanding of the dynamics of immune responses at population level and may guide public health policies.

## Introduction

In many infectious diseases, immunity acquired from infection wanes over time. The same holds for immune responses elicited by vaccines. Typical examples are influenza and COVID-19 where immunity wanes within a few months (Rambhia and Rambhia 2019; Goldberg et al. 2022). The durability of natural immunity and immune responses triggered by vaccines are crucial for decision making and interventions in public health. Antibodies seem to be the protective mechanism for these infections but often more specific immune responses such as specific T cell groups are needed to build up immunity and maintain immune memory. Immunity waning is highly heterogeneous in the population between individuals and changes over time (Lavine et al. 2021).

Several mathematical models have been developed to assess effectiveness and the possibility of waning immunity after infection or vaccination (Montalbán et al. 2022; Iyaniwura et al. 2023; Pell et al. 2022; Gosh et al. 2022; Domenech de Celles et al. 2022; Veliov and Widder 2016). To a lesser extent, the models investigated the optimal timing of vaccine administration, accounting for the waning immunity between seasons for infectious diseases such as influenza (Costantino et al. 2019). A population with heterogeneous immunity is considered in Montalbán et al. (2022). However, individual immunity is modeled as constant over time. In addition, Montalbán et al. (2022) consider no change in immunity levels due to previous infection or vaccination and do not study decision (control) aspects. Iyaniwura et al. (2023) used a distributed delay equations framework to describe the dynamics of waning immunity in a population with vaccine or natural infection induced immunity at an endemic stage. They performed a bifurcation analysis showing that waning immunity from natural infection influences the bifurcation type more than vaccine associated waning immunity. Furthermore, they derived a control reproduction number and showed the interplay between the decrease in immunity rate and the transmission rate of the pathogen. Similar approaches were used by Pell et al. (2022) and Gosh et al. (2022). Domenech de Celles et al. (2022) showed in a simulation study how immunological heterogeneity plays a role in determining the durability of vaccine protection. A model with heterogeneous dynamic immunity where sub-populations were structured with respect to the host immunity was developed and analysed by Veliov and Widder (2016). In all these cases investigation of control aspects was either not present or played a rather limited role.

In Sect. 2 of this study we propose a model that examines the dynamics of an infectious disease, taking into account the waning immunity following natural infection or vaccination. It is designed with the following key considerations:

1. The individuals are heterogeneous with respect to their (dynamic) immunity level;
2. After infection, individual immunity increases progressively until recovery begins;
3. With the onset of recovery, immunity starts to decrease over time;
4. infectiousness, susceptibility, mortality, and recovery rates depend on the individual immunity;
5. The infectiousness of the environment is represented by the aggregated infectiousness of infected individuals, weighted by activity level, as a share of the overall activity level of the population.

The model is formulated in terms of a system of Partial Differential Equations (PDEs) where the latter feature introduces a nonlocal effect in the form of a nonlinear term that incorporates integrals of state variables across the whole range of immunity levels.

From a mathematical perspective, the proposed model is challenging for the following reasons:

(i) It consists of a system of first order PDEs (each of which is of size-structured type, see, e.g., Martcheva and Pilyugin (2006)) with different velocity fields, hence, with different characteristic lines. This creates a substantial problem in the analysis of the system, because a reformulation of the PDE system as an Ordinary Differential Equation (ODE) system in a closed form cannot be obtained.
(ii) Because of the form of the non-local term describing the infectiousness of the environment, the Lipschitz constant of the equations may tend to infinity along the solution, which substantially complicates the proof of global existence.

As previously mentioned, several authors have investigated disease dynamics, considering factors such as waning immunity and the acquisition of immunity during infection or post-vaccination. Similar to our model is the work of White and Medley (1998), which involves equations with different transfer velocities. However, the authors focus on the formal steady-state equations, without examining the overall PDE system. Other studies, such as Rouderfer and Becker (1994), Barbarosa and Röst (2015), Ehrhardt et al. (2019), also consider first order PDEs, but either the velocity fiends are identical or a single PDE (together with ODEs) is involved.

Mathematical features of the model, such as the existence of a solution and the asymptotic behavior, are examined in Sect. 3. The model is then extended in Sect. 4 to encompass effects of vaccination. Additionally, an optimal vaccination problem is formulated in Sect. 4.2, which could potentially be utilized to design vaccine administration strategies.

Finally, in Sect. 5 numerical results are presented for several scenarios, which include the behavior of the epidemic with and without vaccination, as well as optimal vaccination policies. While mathematical properties of the PDE-system are analyzed in some detail, the optimal vaccination problem is analyzed only numerically. Here, analysis focuses on significant qualitative observations regarding the optimal vaccination policy and the corresponding evolution of the epidemic under optimal vaccination.

In the appendix of this work we prove global existence of a solution, even for more general systems than our particular model requires. The proof is not straightforward and may be of independent mathematical interest (see Sect. 3.2 for more explanations).

## The basic model with dynamic immunity

To model the dynamics of the immunity over time, we use a function $\omega : \mathbb {R} \mapsto [0,1]$, whose value, $\omega (t)$, at time *t* is interpreted as the immunity level of an individual at time *t*. The larger this number $\omega (t)$, the higher is the immunity of the individual, which implies lower susceptibility and lower infectiousness. From an empirical point of view, the individual immunity level may be quantified, e.g., proportional the amount of antibodies per *ml* blood.

Throughout the paper, we assume that after an individual is infected, its immunity level increases until the time of recovery (Yaugel-Novoa et al. 2022). Therefore, we describe the evolution of the immunity level after infection at time $\tau$ using the equation

2.1 $$\begin{aligned} \dot{\omega }(t) = g(\omega (t)), \quad \omega (\tau ) = \xi , \quad t \ge \tau , \end{aligned}$$

where $\xi \in [0,1]$ is the initial immunity level at the time of infection $\tau$. The function $g: [0,1] \rightarrow [0, + \infty )$ is assumed to be differentiable and to satisfy $g(0) > 0$, and $g(1) = 0$. The description we use for the immune response to the infectious agent corresponds to the way the immune response may be embedded in a dynamical system that describes the within-host pathogen dynamics of an infection (see Schuh et al. (2023)). In this approach the mounting and decline of the immune responses during an infection process were explicitly captured with an equation describing the overall immune capacity of the individual against the pathogen including the acqusition of an immunity level. In our approach we incorporate these immune response dynamics in an epidemiological model to capture the overall dynamics of the immunity at the population level.^1^

In the long run, the immunity level decreases (Yaugel-Novoa et al. 2022). Therefore, beginning with recovery immunity wanes over time and we describe its decrease by the equation

2.2 $$\begin{aligned} \dot{\omega }(t) = f(\omega (t)), \quad \omega (\tau ) = \xi , \quad t \ge \tau , \end{aligned}$$

where $\xi \in [0,1]$ is the immunity level at the time of recovery $\tau$. It is assumed that $f: [0,1] \rightarrow (- \infty , 0]$, is continuously differentiable with $f(0) = 0$, $f(1) < 0$.

While in a population the individual immunity levels change as described above, at any point in time the individuals in a population may have differing immunity levels, depending on their individual history with the disease. Therefore, the immunity status of a whole population can be modeled as a frequency distribution over the possible values of the immunity level, $\omega \in [0,1]$. Note that $\omega$ here denotes just one possible value of the function $\omega (\cdot )$.

Based on these considerations, we denote by $S(t,\omega )\ge 0$ and $I(t,\omega ) \ge 0$ the size of the susceptible, respectively infected, population of immunity level $\omega$ at time *t*. Thus the total population *N* at time *t* is

$$\begin{aligned} N(t) = \int _0^1 [ S(t,\omega ) + I(t,\omega ) ] \mathrm{\,d}\omega . \end{aligned}$$

In this paper, we assume that the susceptibility and infectiousness of an individual depend only on its immunity level. Immunological memory may have been acquired through a history of previous exposure to the relevant pathogen through infection or vaccination.

The susceptibility is represented by $\sigma (\omega ) \ge 0$, where the continuous function $\sigma : [0,1] \rightarrow [0, \infty )$ is presumably decreasing in $\omega$. Similarly, the infectiousness of infected individuals is expressed by $i(\omega )$, with *i* being a continuous non-negative function.

We denote by $c > 0$ the contact rate of susceptible individuals, while the contact rate of infected individuals is represented by $c_I \in (0,c]$. In principle, the contact parameters can be extended to depend on $\omega$, because people who know that they are well protected by immunity may have more contacts. Moreover, dependence on time may be used for the description of seasonal or other time dependent behaviour of the individuals. However, in this paper, we assume for simplicity that *c* and $c_I$ are constant.

We model the rate of new infections by the expression $c D(t) \sigma (\omega ) S(t, \omega )$. This expresses the fact that the probability of an infection for each susceptible individual is proportional to its contact rate, its susceptibility and the infectiousness of the environment *D*(*t*). For one individual *D* can be considered as a stochastic process, depending on the actions of the individual and on the (random) infectiousness of its contacts. As we are finally interested in a model at the population level and consider classes of population groups with immunity level $\omega$ instead of individuals, we estimate the infectiousness of the environment as an average infectiousness. Infectious individuals of all immunity levels contribute with their infectiousness and at their contact rate to overall infectiousness. Here, the model is based on the mean field idea: for any contact, infectiousness and number of contactees are replaced by a population average.

In order to estimate the probability $\sigma (\omega ) D(t)$ that an individual with immunity level $\omega$ is infected at time *t*, one has also to take into account the contact-adjusted size of the total population at time *t*. Therefore, under the assumption of weighted random mixing, the infectiousness of the environment in which susceptible individuals contact infected individuals is represented as

2.3 $$\begin{aligned} D(t) = \frac{c_I\int _0^1 i(\omega )I(t,\omega ) \mathrm{\,d}\omega }{c_I\int _0^1 I(t,\omega ) \mathrm{\,d}\omega + c \int _0^1 S(t,\omega ) \mathrm{\,d}\omega }. \end{aligned}$$

Finally, the mortality rate of infected individuals is denoted by $\mu (\omega )$, and the recovery rate from infection is denoted by $\rho (\omega )$. Both parameters are nonnegative functions depending on the current immunity level. In the present study we do not model in detail the demographic effects of birth and death rates, which may be even time varying or age-dependent. Neglecting their long-run effects, we basically assume that the birth rate and "natural" death rate are equal, and the same rates are effective for all relevant compartments. The mortality rate $\mu (\omega )$ then represents the excess mortality due to the epidemic.

Based on these assumptions and the related notations, it is now possible to describe the time dependent dynamics of the classes of susceptible and infected individuals for different immunity levels in terms of a system of PDEs for the population sizes *S* and *I* of susceptible and infected individuals with varying immunity level.

2.4 $$\begin{aligned} \frac{\partial }{\partial t} S(t, \omega ) + \frac{\partial }{\partial \omega }(f(\omega ) S(t, \omega ) )= & {} -c D(t) \sigma (\omega ) S(t, \omega ) + \rho (\omega ) I(t,\omega ), \end{aligned}$$

2.5 $$\begin{aligned} \frac{\partial }{\partial t} I(t, \omega ) + \frac{\partial }{\partial \omega }(g(\omega ) I(t, \omega ) )= & {} cD(t) \sigma (\omega ) S(t, \omega ) - (\rho (\omega ) + \mu (\omega )) I(t, \omega ), \nonumber \\ \end{aligned}$$

with initial conditions

2.6 $$\begin{aligned} S(0,\omega ) = S^0(\omega ), \quad I(0,\omega ) = I^0(\omega ), \quad \omega \in [0,1], \end{aligned}$$

($S^0$ and $I^0$ are initial data) and the zero flux boundary conditions

$$\begin{aligned} f(\omega ) S(t, \omega ) = 0, \quad g(\omega )I(t, \omega ) = 0, \quad \omega \in \{0,1\}, \quad t \ge 0. \end{aligned}$$

Due to the assumptions $f(0) = g(1) = 0$ and $f(1) < 0$, $g(0) > 0$, the initial conditions and the zero-flux condition are equivalent to

2.7 $$\begin{aligned} S(t,1) = 0, \quad I(t, 0) = 0, \quad t > 0. \end{aligned}$$

Moreover, due to the meaning of $\omega$, and for consistency of the initial and boundary conditions it is natural to assume that $S^0(1)= I^0(0) = 0$.

In infectious disease epidemiology, modeling by size-structured systems is a well established approach, see e.g. Rouderfer and Becker (1994); White and Medley (1998); Martcheva and Pilyugin (2006); Barbarosa and Röst (2015); Veliov and Widder (2016); Ehrhardt et al. (2019). Each of the equations (2.4) and (2.5) is a standard size-structured equation. The equation represents the evolution of the concentration of a substance moving according to a given velocity field in presence of in- or outflow (the term on the right-hand side). It can be derived by the same (conservation of mass) argument as the advection equation (see e.g. Britton N.F. 1986) for a compressible gas. In contrast with the physical models, in population dynamics such equations usually contain non-local terms (the function *D*(*t*) in our case). Moreover, we deal with a system of two (later three) equations with different transfer velocities, which substantially complicates the analysis.

There is an alternative view of a system represented by equations (2.4), (2.4) (and subsequently, (4.5)), also widely used in mathematical biology. We discuss it in the remaining part of this section, where we normalize the population size such that

$$\begin{aligned} N(0) = 1. \end{aligned}$$

Consequently, the compartment sizes, *S* and *I* (and *V*, in the next section) can be interpreted as proportions of the total population belonging to the respective sub-populations.

At the individual level we basically consider a Markovian stochastic process with hybrid state space: the state of any individual at any given time is characterized by their immune status and the compartment to which they belong at the time. The immune status takes continuous values, while the compartments are finite in number. In particular, compartments are the susceptible, the infected, subsequently also the vaccinated, and the dead individuals. Randomness is introduced only during the discrete transitions between compartments. In the intervals between these transitions, the continuous state progresses according to compartment-specific ordinary differential equations (2.2) and (2.1). Transitions take place in accordance with a Poisson process, with the deceased status serving as an absorbing state for all values of immunity level, and are governed by a time- and state-dependent infinitesimal generator

2.8 $$\begin{aligned} Q(t,\omega ) = \begin{pmatrix} -c D(t) \sigma (\omega ) &{}\quad c D(t) \sigma (\omega ) &{}\quad 0\\ \rho (\omega )&{}\quad -\rho (\omega ) - \mu (\omega )&{}\quad \mu (\omega )\\ 0&{}\quad 0&{}\quad 0 \end{pmatrix} \end{aligned}$$

If we assume for a moment that *D*(*t*) is a given function, the process can be considered as a piecewise deterministic process^2^: in this case we may interpret the proportions $S(t,\omega ),I(t,\omega )$ as probabilities of being in the susceptible or infected state with immunity $\omega$ at time *t*. Then, equations (2.4)-(2.5), augmented by

2.9 $$\begin{aligned} \frac{\partial }{\partial t} G(t, \omega ) = \mu (\omega ) I(t, \omega ), \end{aligned}$$

where $G(t, \omega )$ denotes the “probability” of being dead at time *t* (and having died at immunity level $\omega$) is the Fokker-Planck (or Kolmogorov forward) equation of the process, see e.g., Annunziato and Borzi (2018).

The standard class of piecewise deterministic processes, sometimes also called "correlated random walk", was introduced by Davis (1984) and has been used extensively in theoretical biology, see, e.g., Rudnicki and Tyran-Kamińska (2000). Our model extends the standard piecewise deterministic case because *D*(*t*) is defined by (2.3), involving integrals over $\omega$ in a nonlinear way. Basically, the jump rate from *S* to *I* depends on the distribution of the whole population over all relevant compartments and all values of immunity levels at any point in time.

## Existence of solution and asymptotic behaviour

### Notion of solution

The following assumptions hold throughout the paper.

*Standing Assumptions.* The functions *f* and *g* are differentiable with Lipschitz derivatives, defined in a neighborhood of [0, 1], with $f(0) = g(1) = 0$, and the derivatives $f'(\omega ) < 0$ on (0, 1], $g'(\omega ) < 0$ in $\omega \in [0,1)$. The function $i: [0,1] \rightarrow [0, \infty )$ is measurable and bounded, the functions $\sigma ,\rho , \mu , S^0, I^0: [0,1] \rightarrow [0, \infty )$ are continuous, differentiable on (0, 1) except for a finite number of points,^3^ and the derivatives are Lipschitz continuous in each interval of existence. Moreover, $S^0(1) = I^0(0) = 0$ and $\int _0^1 (S^0(\omega ) + I^0(\omega ) )\mathrm{\,d}\omega = 1$.

Solution of a system (2.3)–(2.7) may be defined in several ways (cf. Kato and Torikata (1997)). Here, we define the notion of solution by the method of characteristics. For reasons of further analysis, we restrict the definition to the case of Lipschitz continuous solutions (although the solutions may be discontinuous for general initial or boundary data).

Denote $\Gamma := [0,T] \times [0,1]$, and let $\tilde{\Gamma }\subset \mathbb {R}^2$ be an open neighborhood of $\Gamma$. For $\gamma := (\tau ,\xi ) \in \tilde{\Gamma }$ we denote by $\omega ^f[\gamma ](\cdot )$ and $\omega ^g[\gamma ](\cdot )$ the solutions of (2.2) and (2.1), respectively. Due to the assumptions for *f* and *g*, the set [0, 1] is an invariant domain for both equations; hence, considering a sufficiently small neighborhood $\tilde{\Gamma }$ of $\Gamma$, the solutions are defined on [0, *T*] for every $\gamma \in \tilde{\Gamma }$.

Further, denote $\Gamma _f:= (\{0\} \times [0,1]) \cup ([0,T] \times \{1\})$ (the left-upper boundary of $\Gamma$), $\Gamma _g:= (\{0\} \times [0,1]) \cup ([0,T] \times \{0\})$ (the left-lower boundary of $\Gamma$). Due to the assumptions for *f* and *g*, we have that $\cup _{\gamma \in \Gamma _f} \, \omega ^f[\gamma ](t) = [0,1]$. Similarly, $\cup _{\gamma \in \Gamma _g} \, \omega ^g[\gamma ](t) = [0,1]$. Again due to the properties of *f* and *g*, there are unique functions $\gamma ^f: \tilde{\Gamma }\rightarrow \Gamma _f$ and $\gamma ^g: \tilde{\Gamma }\rightarrow \Gamma _g$ such that $\omega ^f[\gamma ^f(t,\omega )](t) = \omega$ and $\omega ^g[\gamma ^g(t,\omega )](t) = \omega$ for all $\tilde{\Gamma }$. Moreover, due to the (Lipschitz) continuous dependence of the solutions of (2.2) and (2.1) on the initial data, the functions $\omega ^f, \omega ^g, \gamma ^f, \gamma ^g$ have Lipschitz continuous derivatives with respect to $\gamma$ and *t*.

**Fig. 1:**
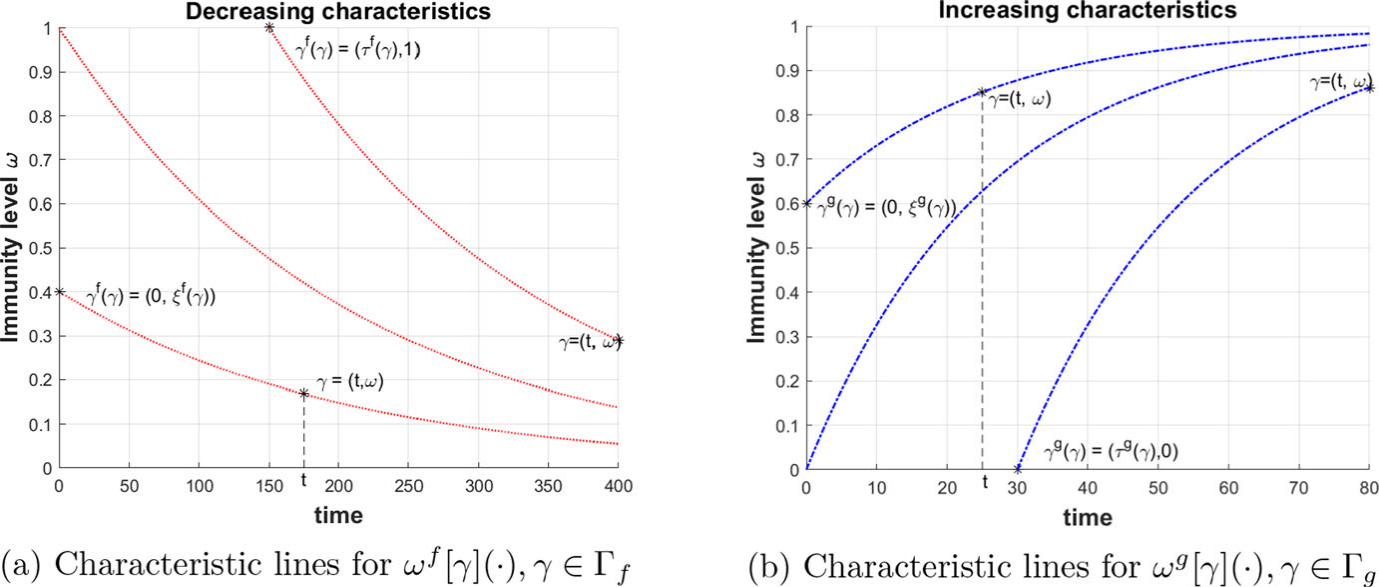
Characteristic lines and illustration of the notations

For the (dummy) real numbers $t,\omega ,d, s,i$, denote (in relation to (2.4)–(2.5))

3.1 $$\begin{aligned} F^S(t,\omega ,d, s,i):= & {} -c d \sigma (\omega ) s + \rho (\omega ) i - f'(\omega ) s, \end{aligned}$$

3.2 $$\begin{aligned} F^I(t,\omega ,d, s,i):= & {} c d \sigma (\omega ) s - (\rho (\omega ) + \mu (\omega )) i - g'(\omega ) i, \end{aligned}$$

where the argument *t* is included for further use. For shortness we introduce the notations

$$\begin{aligned} \gamma ^f(\gamma ):= (\tau ^f(\gamma ), \xi ^f(\gamma )), \quad \gamma ^g(\gamma ):= (\tau ^g(\gamma ), \xi ^g(\gamma )), \end{aligned}$$

for $$\gamma \in \Gamma $$, and

$$\begin{aligned} {\bar{S}}^0(\gamma ):= \left\{ \begin{array}{cl} S^0(\xi ) &{} \text{ if } \gamma = (0,\xi ), \\ 0 &{} \text{ if } \gamma = (\tau ,1), \end{array} \right. \qquad {\bar{I}}^0(\gamma ):= \left\{ \begin{array}{cl} I^0(\xi ) &{} \text{ if } \gamma = (0,\xi ), \\ 0 &{} \text{ if } \gamma = (\tau ,0). \end{array} \right. \end{aligned}$$

for $\gamma \in \Gamma _f$ and $\gamma \in \Gamma _g$, respectively. Figure 1 illustrates these notations.

The so-called “characteristic lines” staring from $\Gamma _f$ cover $\Gamma$, more precisely, the mapping $\{ \gamma \in \Gamma _f, \, t \in [\tau ^f(\gamma ),T] \} \ni (\gamma ,t) \mapsto (t,\omega ^f[\gamma ](t)) \in \Gamma$ is bijective. A similar fact applies to the characteristic lines emanating from $\Gamma _g$. Then any pair of continuous functions $(S,I): \Gamma \rightarrow \mathbb {R}^2$ uniquely determines two family of functions of *t* parameterized by $\gamma$:

3.3 $$\begin{aligned} z^S[\gamma ](t):= & {} S(t,\omega ^f[\gamma ](t)), \quad \gamma \in \Gamma _f, \, t \in [\tau ^f(\gamma ),T] \end{aligned}$$

3.4 $$\begin{aligned} z^I[\gamma '](t):= & {} I(t,\omega ^g[\gamma '](t)), \quad \gamma ' \in \Gamma _g, \, t \in [\tau ^g(\gamma '),T]. \end{aligned}$$

Vice versa, any pair of continuous functions $(z^S[\cdot ](\cdot ),z^I[\cdot ](\cdot ))$ defined on the sets as in the previous exposed lines determines a continuous pair $(S,I): \Gamma \rightarrow \mathbb {R}^2$ by the relations

$$\begin{aligned} S(t,\omega ):= z^S[\gamma ^f(t,\omega )](t), \quad I(t,\omega ):= z^I[\gamma ^g(t,\omega )](t). \end{aligned}$$

These facts explain the following definition.

#### Definition 3.1

The pair of continuous functions $S, I: \Omega \rightarrow \mathbb {R}$ is called a solution of system (2.3)–(2.7) if the functions $z^S$ and $z^{I}$ defined by (3.3)–(3.4) are absolutely continuous in *t* and satisfy the equations

3.5 $$\begin{aligned}{} & {} \dot{z}^S[\gamma ](t) = F^S(t, \omega ^f[\gamma ](t),D(t), z^S[\gamma ](t), I(t,\omega ^f[\gamma ](t))), \nonumber \\{} & {} \quad \quad \gamma \in \Gamma _f, \; t \in [\tau ^f(\gamma ),T] \end{aligned}$$

3.6 $$\begin{aligned}{} & {} \dot{z}^I[\gamma '](t) = F^I(t, \omega ^g[\gamma '](t),D(t), S(t,\omega ^g[\gamma '](t)), z^I[\gamma '](t)), \nonumber \\{} & {} \quad \gamma ' \in \Gamma _g, \; t \in [\tau ^g(\gamma '),T], \end{aligned}$$

together with (2.3) and with initial conditions

$$\begin{aligned} z^S[\gamma ](\tau ^f(\gamma )) = {\bar{S}}_0(\gamma ), \quad z^I[\gamma '](\tau ^g(\gamma ')) = {\bar{S}}_0(\gamma '). \end{aligned}$$

Equivalently, for any $\gamma = (t,\omega ) \in \Gamma$ it holds that

3.7 $$\begin{aligned} S(\gamma )= & {} \int _{\tau ^f(\gamma )}^t F^S\big (s,\omega ^f[\gamma ](s), D(s), S(s,\omega ^f[\gamma ](s)),I(s,\omega ^f[\gamma ](s))\big ) \mathrm{\,d}s + {\bar{S}}^0(\gamma ^f(\gamma )), \nonumber \\ \end{aligned}$$

3.8 $$\begin{aligned} I(\gamma )= & {} \int _{\tau ^g(\gamma )}^t F^I\big (s,\omega ^g[\gamma ](s), D(s), S(s,\omega ^g[\gamma ](s)),I(s,\omega ^g[\gamma ](s))\big ) \mathrm{\,d}s + {\bar{I}}^0(\gamma ^g(\gamma )). \nonumber \\ \end{aligned}$$

#### Remark 3.1

We mention that if *S* and *I* are differentiable and satisfy equations (2.3)–(2.7) in the classical sense, then the corresponding representations $(z^S[\cdot ](\cdot ),z^I[\cdot ](\cdot ))$ on the characteristic lines solve equations (3.3), (3.4). Moreover, the representation (3.7), (3.8) is valid. The latter fact is not straightforward, however it can be directly checked using the identities $\frac{\partial }{\partial t} \omega ^f[\gamma ](s) + f(\omega ) \frac{\partial }{\partial \omega } \omega ^f[\gamma ](s) = 0$ for all $\gamma = (t,\omega ) \in \Gamma _f$, and a few more similar identities that appear when plugging the expressions of *S* and *I* in (3.7)–(3.8) into (2.4)–(2.5).

### Existence of a “smooth" solution

In this subsection we present a theorem claiming existence of a solution of system (2.3)–(2.7) which is regular enough to enable the subsequent analysis. Although the proof is based on the Banach contraction mapping theorem, it is not straightforward due to two reasons: (i) the Lipschitz constants of $F^S$ and $F^{I}$ may tend to infinity with the time due to the expression (2.3) for *D*, which makes the existence on $[0,\infty )$ problematic; (ii) due to the involvement of different transfer velocity fields *f* and *g*, the system (2.4)–(2.5) cannot be reduced to a closed form ODE system along the characteristics; (iii) proving the non-negativity of the solution is not straightforward at all. Therefore, we present in the Appendix a detailed proof of the existence theorem formulated below. In fact, we even prove a more general theorem assuming a few properties of the functions $F^S$ and $F^{I}$ in (3.7)–(3.8) and not necessarily the specific form of (3.1)–(3.2).

#### Theorem 3.1

Under the standing assumptions, system (2.3)–(2.7) has a unique solution (*S*, *I*) on $[0, \infty )\times [0,1]$ which is Lipschitz continuous on every set $[0,T] \times [0,1]$, $T > 0$. The solution is nonnegative and satisfies $\int _0^1 [S(t,\omega ) + I(t,\omega )] \mathrm{\,d}\omega > 0$ for all $t \in [0,\infty )$. Moreover, for each $\omega \in [0,1]$ and $T \in (0,\infty )$ the derivatives $\frac{\partial }{\partial t} S(t, \omega ), \frac{\partial }{\partial t} I(t, \omega )$ exist on (0, *T*] except of finite number of points, and for each $t \in (0,\infty )$ the derivatives $\frac{\partial }{\partial \omega } S(t, \omega ), \frac{\partial }{\partial \omega } I(t, \omega )$ exist on (0, 1) except of finite number of points.

Including more equations with different characteristic curves (such as the system with vaccination in the next section) does not change the proof. Since we allow dependence of the functions $F^S$ and $F^{I}$ on time in the proof, the presence of a control function in the equations is also covered by the existence theorem as proved in the Appendix.

The differentiability property in the claim of the theorem is crucial: it not only enables integration of equations (2.4)–(2.5) with respect to $\omega$ but also permits to interchange the order of integration and differentiation. This, in turn, implies that the aggregated compartment sizes

$$\begin{aligned} {\hat{S}}(t):= \int _0^1 S(t,\omega ) \mathrm{\,d}\omega , \qquad {\hat{I}}(t):= \int _0^1 I(t,\omega ) \mathrm{\,d}\omega . \end{aligned}$$

can be represented as in the following corollary.

#### Corollary 3.2

Let (*S*, *I*, *D*) be the solution of (2.3)–(2.7) on $[0,\infty )\times [0,1]$. Then the aggregated compartment sizes ${\hat{S}}(t),\, {\hat{I}}(t)$ are given by

3.9 $$\begin{aligned} {\hat{S}}(t)\!=\! & {} \int _0^1 S^0(\omega ) \mathrm{\,d}\omega -c \int _0^t D(t) \int _0^1 \sigma (\omega ) S(t, \omega ) \mathrm{\,d}\omega \mathrm{\,d}t + \int _0^t \int _0^1 \rho (\omega ) I(t,\omega ) \mathrm{\,d}\omega \mathrm{\,d}t,\nonumber \\ \end{aligned}$$

3.10 $$\begin{aligned} \!\!\!\!\!\!{\hat{I}}(t)\!=\! & {} \int _0^1 I^0(\omega ) \mathrm{\,d}\omega + c \int _0^t D(t) \int _0^1 \sigma (\omega ) S(t, \omega ) \mathrm{\,d}\omega \mathrm{\,d}t - \int _0^t \int _0^1(\rho (\omega ) + \mu (\omega )) I(t,\omega ) \mathrm{\,d}\omega \mathrm{\,d}t. \nonumber \\ \end{aligned}$$

In addition, the total number of individuals decreases according to

3.11 $$\begin{aligned} N(t) = {\hat{S}}(t) + {\hat{I}}(t) = - \int _0^1 \mu (\omega ) I(t,\omega ) \mathrm{\,d}\omega . \end{aligned}$$

#### Proof

Integrate equations (6.1)–(2.5) over $\omega \in [0,1]$ and exchange the order of integrals and differentials on the left hand side. This is possible due to the properties of *S* and *I* in Theorem 3.1. Apply the zero flux conditions (specified after (2.6)), to obtain expressions for $\frac{\mathrm{\,d}{\hat{S}}(t)}{\mathrm{\,d}t}$ and $\frac{\mathrm{\,d}{\hat{I}}(t)}{\mathrm{\,d}t}$. These derivatives exist for all *t* except for a finite number of points on every bounded set [0, *T*]. Finally, integrate over $t\in [0,1]$ to obtain equations (3.9)–(3.10). Equation (3.11) is then obtained by adding up. $\square$

### Descend of the epidemics and basic reproduction numbers

The goal of this subsection is to obtain conditions under which the number of infected individuals decreases and converges to a disease-free state. We proceed under the assumption that the conditions stipulated in Theorem 3.1 are satisfied and introduce the notation

$$\begin{aligned} \dot{{\hat{S}}}(t):= \frac{\mathrm{\,d}{\hat{S}}(t)}{\mathrm{\,d}t} \text { and } \dot{{\hat{I}}}(t):= \frac{\mathrm{\,d}\hat{I}(t)}{\mathrm{\,d}t}. \end{aligned}$$

Using the estimation

3.12 $$\begin{aligned} c D(t) \le \frac{c_I \int _0^1 i(\omega ) I(t,\omega ) \mathrm{\,d}\omega }{{\hat{S}}(t)}, \end{aligned}$$

which can easily be derived from the definition (2.3), we obtain by differentiating equation (3.10)

$$\begin{aligned} \dot{{\hat{I}}}(t) \le \frac{c_I \int _0^1 i(\omega ') I(t,\omega ') \mathrm{\,d}\omega '}{{\hat{S}}(t)} \int _0^1 \sigma (\omega ) S(t,\omega ) \mathrm{\,d}\omega - \int _0^1 (\rho (\omega ) + \mu (\omega ))] I(t,\omega ) \mathrm{\,d}\omega , \end{aligned}$$

hence,

3.13 $$\begin{aligned} \dot{{\hat{I}}}(t) \le \int _0^1 \big [c_I i(\omega ) \int _0^1 \sigma (\omega ') \frac{S(t,\omega ')}{{\hat{S}}(t)} \mathrm{\,d}\omega ' - (\rho (\omega ) + \mu (\omega )) \big ] \frac{I(t,\omega )}{{\hat{I}}(t)} \mathrm{\,d}\omega {\hat{I}}(t).\nonumber \\ \end{aligned}$$

In two steps we eliminate the dependence of the estimation on the densities of *S* and *I*, focusing on the worst case:

3.14 $$\begin{aligned}&\displaystyle \dot{{\hat{I}}}(t) \le \int _0^1 [c_I \bar{\sigma }i(\omega ) - (\rho (\omega ) + \mu (\omega ))] \frac{I(t,\omega )}{{\hat{I}}(t)} \mathrm{\,d}\omega \hat{I}(t), \qquad \bar{\sigma }:= \sup \{ \sigma (\omega ): \, \omega \in [0,1]\}, \nonumber \\ \end{aligned}$$

3.15 $$\begin{aligned}&\displaystyle \dot{{\hat{I}}}(t) \le \int _0^1 \max _{\omega \in [0,1]} \{c_I \bar{\sigma }i(\omega ) - (\rho (\omega ) + \mu (\omega ))\} \mathrm{\,d}\omega {\hat{I}}(t). \end{aligned}$$

Define the numbers

$$\begin{aligned} \lambda _t:= & {} \int _0^1 \big [\rho (\omega ) + \mu (\omega ) - c_I i(\omega ) \int _0^1 \sigma (\omega ') \frac{S(t,\omega ')}{{\hat{S}}(t)} \mathrm{\,d}\omega ' \big ] \frac{I(t,\omega )}{{\hat{I}}(t)}\mathrm{\,d}\omega ,\\ \lambda:= & {} \min _{\omega \in [0,1]} \{\rho (\omega ) + \mu (\omega ) - c_I \bar{\sigma }i(\omega )\}. \end{aligned}$$

Obviously $\lambda _t \ge \lambda$ for any $t \ge 0$. Thus we obtain the following proposition.

#### Proposition 3.3

At any time *t*, if the current normalized densities of susceptible and infected individuals, $S(t,\cdot )/{\hat{S}}(t)$ and $I(t,\cdot )/{\hat{I}}(t)$, satisfy the inequality $\lambda _t > 0$ then the number of infected individuals strictly decreases at this time. Moreover,

3.16 $$\begin{aligned} {\hat{I}}(t) \le e^{-\lambda (t - \tau )} {\hat{I}}(\tau ), \qquad 0 \le \tau \le t, \quad k = 1,2,3. \end{aligned}$$

The next corollary claims that the susceptible population does not asymptotically extinct. Furthermore, it provides an estimate of the maximum population reduction attributable to the disease.

#### Corollary 3.4

Assume that $\lambda > 0$ and denote $b:= \max \{ 0, \max _{\omega \in [0,1]} \{c_I \bar{\sigma }i(\omega ) - \rho (\omega )\} \}$ Then for any initial state $(S^0(\cdot ),I^0(\cdot ))$ satisfying $\int _0^1 I^0(\omega ) \mathrm{\,d}\omega < \frac{\lambda }{\lambda + b}$, the susceptible population size ${\hat{S}}(t)$ satisfies

$$\begin{aligned} {\hat{S}}(t) \ge {\hat{S}}(0) - \frac{b}{\lambda + b} > 0, \qquad t \ge 0. \end{aligned}$$

#### Proof

From equation (3.9) and (3.12) we have

$$\begin{aligned} \begin{aligned} \dot{\hat{S}}(t)&=-c D(t) \int _0^1 \sigma (\omega ) S(t, \omega ) d \omega +\int _0^1 \rho (\omega ) I(t,\omega ) \mathrm{\,d}\omega \\&\ge -c D(t)\bar{\sigma }\hat{S}(t)+\int _0^1 \rho (\omega ) I(t, \omega ) \mathrm{\,d}\omega \\&\ge \int _0^1\left( -c_I i(\omega )+\rho (\omega )\right) I(t, \omega ) \mathrm{\,d}\omega \ge -b \hat{I}(t), \end{aligned} \end{aligned}$$

because $$c_I i(w)-\rho (\omega ) \le b$$, $$\omega \in [0,1]$$. Then,

$$\begin{aligned} \begin{aligned} \hat{S}(t)&=\hat{S}(0)+\int _0^t \dot{\hat{S}}(s) \mathrm{\,d}s \geqslant \hat{S}(0)-b \int _0^t \hat{I}(s) \mathrm{\,d}s \\&\geqslant \hat{S}(0)-b \int _0^t e^{-\lambda s} \hat{I}(0) \mathrm{\,d}s \geqslant \hat{S}(0)-\frac{b}{\lambda } \hat{I}(0) \\&\geqslant \hat{S}(0)-\frac{b}{\lambda } \frac{\lambda }{\lambda +b}=\hat{S}(0)-\frac{b}{\lambda +b}>0 \end{aligned} \end{aligned}$$

The last inequality follows from

$$\begin{aligned} \hat{S}(0)=1-\hat{I}(0)\ge 1-\frac{\lambda }{\lambda +b}=\frac{b}{\lambda +b}. \end{aligned}$$

$$\square $$

We mention that in absence of disease, i.e. $I^0(\cdot ) = 0$, the density of the susceptible individuals converges to the Dirac delta function concentrated at zero, irrespectively of the starting distribution $S(t,\omega )$. To formally show this fact we consider the purely susceptible population starting from $S^0(\omega )$ with $\int _0^1 S^0(\omega ) \mathrm{\,d}\omega = 1$. Then $S(t,\omega )$ and $I(t,\omega ) = 0$ solves (2.4)–(2.5), where *S* is the solution of

$$\begin{aligned} \frac{\partial }{\partial t} S(t, \omega ) + \frac{\partial }{\partial \omega }(f(\omega ) S(t, \omega ) ) = 0 \end{aligned}$$

with $S(0,\omega ) = S^0(\omega )$ and $S(t,1) = 0$. Solving the last equation along the characteristics, after some elementary calculation we show that for every $\varphi \in L_\infty (0,1)$

$$\begin{aligned} \int _0^1 S(t,\omega ) \varphi (\omega ) \mathrm{\,d}\omega = \int _0^1 S^0(\xi ) \varphi (\omega ^f[0,\xi ](t)) \mathrm{\,d}\xi . \end{aligned}$$

Let $\varphi (\omega ) = 1$ on $[0,\varepsilon ]$, else $\varphi (\omega ) = 0$. Due to the properties of *f* we have that $\omega _f[0,\xi ](t) \le \omega [0,1](t)$ and there is $t(\varepsilon )$ such that for any $t \ge t(\varepsilon )$ we have $\omega ^f[0,1](t) \le \varepsilon$. Then

$$\begin{aligned} \int _0^\varepsilon S(t,\omega ) \mathrm{\,d}\omega = \int _0^1 S(t,\omega ) \varphi (\omega ) \mathrm{\,d}\omega = \int _0^1 S^0(\xi ) \varphi (\omega ^f[0,\xi ](t)) \mathrm{\,d}\xi = \int _0^1 S^0(\xi ) \mathrm{\,d}\xi = 1 \end{aligned}$$

for $t \ge t(\varepsilon )$. In particular, $S(t,\omega ) = 0$ for a.e. $\omega \in [\varepsilon ,1]$ for such *t*. Now, take an arbitrary $\varphi \in W^{1,\infty }$ (the domain is [0, 1], therefore $W^{1,\infty }$ is the set of all Lipschitz continuous functions with the usual norm). We can estimate (using the obvious relation $\int _0^1 S(t,\omega ) \mathrm{\,d}\omega = 1$)

$$\begin{aligned} \int _0^1 S(t,\omega ) \varphi (\omega ) \mathrm{\,d}\omega = \varphi (0) + \int _0^1 S(t,\omega ) [\varphi (\omega ) - \varphi (0)] \mathrm{\,d}\omega . \end{aligned}$$

For $t \ge t(\varepsilon )$ the last term can be estimated by

$$\begin{aligned} \int _0^\varepsilon S(t,\omega ) [\varphi (\omega ) - \varphi (0)] \mathrm{\,d}\omega\le & {} \Vert \varphi ' \Vert _\infty \int _0^\varepsilon S(t,\omega ) \omega \mathrm{\,d}\omega \\\le & {} \varepsilon \Vert \varphi ' \Vert _\infty \int _0^1 S(t,\omega ) \mathrm{\,d}\omega = \varepsilon \Vert \varphi ' \Vert _\infty . \end{aligned}$$

We can define the Dirac $\delta$-function as an element of the dual space $(W^{1,\infty })^*$, and we can also view $S(t,\cdot )$ as such. Then

$$\begin{aligned} \big | \int _0^1 S(t,\omega ) \varphi (\omega ) \mathrm{\,d}\omega - \delta _0 \varphi \big | \le \varepsilon \Vert \varphi \Vert _{W^{1,\infty }}. \end{aligned}$$

for $t \ge t(\varepsilon )$, which means that $S(t,\cdot ) \longrightarrow \delta _0$ with $t \rightarrow +\infty$ in the norm of $(W^{1,\infty })^*$.

**Basic reproduction numbers.** Next, we investigate the related issue of basic reproduction number. For this, we consider a “small” portion of infected individuals, $I^0(\omega )$, $\omega \in [0,1]$, inserted in the susceptible population $S^0(\omega )$, $\omega \in [0,1]$, with $\int _0^1 (S^0(\omega ) + I^0(\omega ) ) \mathrm{\,d}\omega = 1$. This initially infected population changes over time due to recovery, mortality and increased immunity level, according to the equation

$$\begin{aligned} \frac{\partial }{\partial t} I(t, \omega ) + \frac{\partial }{\partial \omega }(g(\omega ) I(t, \omega ) ) = - (\rho (\omega ) + \mu (\omega )) I(t, \omega ), \end{aligned}$$

with side conditions $I(0,\omega ) = I^0(\omega )$, $I(t,0) = 0$. The solution, call it $I^0(t,\omega )$ (in the same sense as defined in the previous subsections), can be presented as

$$\begin{aligned} I^0(t,\omega ) = \left\{ \begin{array}{cl} 0 &{} \text{ if } \omega \in [0, \omega ^g[0,0](t)), \\ y[\xi ](t) &{} \text{ if } \omega = \omega ^g[0,\xi ](t) \in [\omega ^g[0,0](t),1], \, \xi \in [0,1], \end{array} \right. \end{aligned}$$

where $y[\xi ](\cdot )$ is the solution of the ODE (along the characteristic line $\omega = \omega ^g[0,\xi ](t)$)

$$\begin{aligned}{} & {} \dot{y}[\xi ](t) = -(\rho (\omega ^g[0,\xi ](t)) + \mu (\omega ^g[0,\xi ](t)))y[\xi ](t) - g'(\omega ^g[0,\xi ](t))y[\xi ](t),\\{} & {} \quad y[\xi ](0) = I^0(\xi ). \end{aligned}$$

Thus, abbreviating $$\kappa := \rho + \mu $$,

$$\begin{aligned} y[\xi ](t) = e^{- \int _0^t (\kappa (\omega ^g[0,\xi ](s)) + g'(\omega ^g[0,\xi ](s))) \mathrm{\,d}s}\, I^0(\xi ). \end{aligned}$$

Below we need the linearization of the function *D* in (2.3) with respect to $I=I^0$ at $I = 0$ and $S^0$, which is $c_I \int _0^1 i(\omega ') I^0(t,\omega ') \mathrm{\,d}\omega '$. Following the terminology in Diekmann and Heesterbeek (2000), we represent the “next generation” of infected individuals resulting from the “small” group of initially infected population $I^0(\omega )$ as the solution of the equation

$$\begin{aligned}{} & {} \frac{\partial }{\partial t} I(t, \omega ) + \frac{\partial }{\partial \omega }(g(\omega ) I(t, \omega ) ) = c_I \int _0^1 i(\omega ') I^0(t,\omega ') \mathrm{\,d}\omega ' \,\sigma (\omega ) S^0(\omega ),\\{} & {} \qquad I(0,\omega ) = 0, \;\; I(t,0) = 0. \end{aligned}$$

Integrating in $\omega$ (see Corollary 3.2 and its proof), we obtain for ${\hat{I}}(t):= \int _0^1 I(t,\omega ) \mathrm{\,d}\omega$ the expression

$$\begin{aligned} \dot{{\hat{I}}}(t)= & {} c_I \int _0^1 i(\omega ') I^0(t,\omega ') \mathrm{\,d}\omega ' \, \int _0^1 \sigma (\omega ) S^0(\omega ) \mathrm{\,d}\omega , \\= & {} c_I \int _{\omega ^g[0,0](t)}^1 i(\omega ') I^0(t,\omega ') \mathrm{\,d}\omega ' \, \int _0^1 \sigma (\omega ) S^0(\omega ) \mathrm{\,d}\omega , \quad {\hat{I}}(0) = 0. \end{aligned}$$

Changing the variable $\omega ' = \omega ^g[0,\xi ](t)$ and substituting $I^0$ with $y[\xi ]$ we obtain the expression

$$\begin{aligned} \dot{{\hat{I}}}(t) = c_I \int _0^1 i(\omega ^g[0,\xi ](t)) \,y[\xi ](t) \, \frac{\partial }{\partial \xi } \omega ^g[0,\xi ](t) \mathrm{\,d}\xi \, \int _0^1 \sigma (\omega ) S^0(\omega ) \mathrm{\,d}\omega . \end{aligned}$$

Since by a standard argument $\frac{\partial }{\partial \xi } \omega ^g[0,\xi ](t) = e^{\int _0^t g'(\omega ^g[0,\xi ](s) \mathrm{\,d}s}$, we obtain that

$$\begin{aligned} \dot{{\hat{I}}}(t) = c_I \int _0^1 \sigma (\omega ) S^0(\omega ) \mathrm{\,d}\omega \, \int _0^1 i(\omega ^g[0,\xi ](t)) e^{- \int _0^t \kappa (\omega ^g[0,\xi ](s)) \mathrm{\,d}s} \, I^0(\xi ) \mathrm{\,d}\xi . \end{aligned}$$

Integrating in *t* we obtain the expression

$$\begin{aligned} {\hat{I}}(\infty ){} & {} = c_I \int _0^1 \sigma (\omega ) S^0(\omega ) \mathrm{\,d}\omega \, \int _0^1 \int _0^\infty i(\omega ^g[0,\xi ](t)) e^{- \int _0^t \kappa (\omega ^g[0,\xi ](s)) \mathrm{\,d}s} \\{} & {} \quad \mathrm{\,d}t \frac{ I^0(\xi )}{\int _0^1 I^0(\omega ) \mathrm{\,d}\omega } \mathrm{\,d}\xi \, \int _0^1 I^0(\omega ) \mathrm{\,d}\omega . \end{aligned}$$

The left-hand side represents the total amount of secondary infections directly caused by $I^0$, while the last multiplier on the right is the total amount of initially infected. Thus we can define the basic reproduction number of the disease, under the assumption that exact information about the $\omega$-density of the initially infected and the susceptible population:

$$\begin{aligned} {{\mathcal {R}}}_0[S^0(\cdot ), I^0(\cdot )]= & {} c_I \int _0^1 \sigma (\omega ) S^0(\omega ) \mathrm{\,d}\omega \, \int _0^1 \int _0^\infty i(\omega ^g[0,\xi ](t))\, e^{- \int _0^t \kappa (\omega ^g[0,\xi ](s)) \mathrm{\,d}s} \\{} & {} \quad \mathrm{\,d}t \, \frac{ I^0(\xi )}{\int _0^1 I^0(\omega ) \mathrm{\,d}\omega } \mathrm{\,d}\xi . \end{aligned}$$

We mention that the above improper integral may diverge. Clearly, a natural sufficient condition for convergence is that $\mu (1) + \rho (1) > 1$.

Changing the variable *t* with $\omega = \omega ^g[0,\xi ](t)$ and the variable *s* with $\eta = \omega ^g[0,\xi ](s)$ we obtain the equivalent formula

$$\begin{aligned} {{\mathcal {R}}}_0[S^0(\cdot ), I^0(\cdot )] = c_I \int _0^1 \sigma (\omega ) S^0(\omega ) \mathrm{\,d}\omega \, \int _0^1 \int _\xi ^1 \frac{i(\omega )}{g(\omega )}\, e^{- \int _\xi ^\omega \frac{\kappa (\eta )}{g(\eta )} \mathrm{\,d}\eta } \mathrm{\,d}\omega \, \frac{ I^0(\xi )}{\int _0^1 I^0(\omega ) \mathrm{\,d}\omega } \mathrm{\,d}\xi . \end{aligned}$$

If exact information about the $\omega$-density of the initially infected individuals is not available, a worst case scenario is included in the definition, as it is usual in the considerations of the basic reproduction number (see e.g. Von den Driessche and Watmough 2008). If only the density of $S^0$ is known considering the worst case of $\frac{ I^0(\xi )}{\int _0^1 I^0(\omega ) \mathrm{\,d}\omega }$ gives the expression

$$\begin{aligned} {\hat{I}}(\infty ) \le c_I \int _0^1 \sigma (\omega ) S^0(\omega ) \mathrm{\,d}\omega \, \mathcal{M}\, \int _0^1 I^0(\omega ) \mathrm{\,d}\omega , \end{aligned}$$

where

$$\begin{aligned} \mathcal{M}:= \max _{\xi \in [0,1]} \int _0^\infty i(\omega ^g[0,\xi ](t) \, e^{- \int _0^t \kappa (\omega ^g[0,\xi ](s)) \mathrm{\,d}s} \mathrm{\,d}t \,=\, \max _{\xi \in [0,1]} \int _\xi ^1 \frac{i(\omega )}{g(\omega )}\, e^{- \int _\xi ^\omega \frac{\kappa (\eta )}{g(\eta )} \mathrm{\,d}\eta } \mathrm{\,d}\omega . \end{aligned}$$

This leads to the definition of basic reproduction number of the disease, under the assumption that exact information about the $\omega$-density of susceptible population is known:

$$\begin{aligned} {{\mathcal {R}}}_0[S_0(\cdot )] = c_I \int _0^1 \sigma (\omega ) S^0(\omega ) \mathrm{\,d}\omega \, \mathcal{M}. \end{aligned}$$

Finally, if no exact information about the initial density of the susceptible population is available, then the worst case scenario gives the basic reproduction number

$$\begin{aligned} {{\mathcal {R}}}_0 = c_I \mathcal{M}\,\max _\omega \sigma (\omega ). \end{aligned}$$

Notice that one can estimate ${\mathcal R}_0[S_0(\cdot )]$ and ${{\mathcal {R}}}_0$ using the obvious inequality

$$\begin{aligned} \mathcal{M}\le \frac{\max _\omega i(\omega )}{ \min _\omega \kappa (\omega )}. \end{aligned}$$

In particular, this gives

3.17 $$\begin{aligned} {{\mathcal {R}}}_0 \le c_I \max _\omega \sigma (\omega ) \frac{\max _\omega i(\omega )}{ \min _\omega (\rho (\omega ) + \mu (\omega ))}. \end{aligned}$$

For comparison, we mention that in the case of data independent of $\omega$, the estimation of basic reproduction number (3.17) reduces to $\frac{c_I i \sigma }{\rho +\mu }$, which coincides with the usual expression for the basic reproduction in the SIR model.

## Modeling and optimization of vaccination

In this section we introduce a control variable that represents the vaccination efforts and consider a class of optimization problems for the vaccination policy.

### Modelling vaccination

Including vaccination requires an extension of the basic model 2.3–2.7. We assume that only susceptible individuals are vaccinated. It is necessary then to model the act of vaccination together with the immunological behavior of vaccinated individuals.

We denote by $v(t,\omega )$ the vaccination rate applied to susceptible individuals of immunity level $\omega$ at time *t*. This means, that $v(t,\omega )S(t,\omega )$ individuals of immunity level $\omega$ become vaccinated at time *t*.

The effect of vaccination on immunity is not immediate. Like newly infected individuals, vaccinated individuals gain immunity over time, until their immunity level reaches a maximum, possibly depending on the immunity level before vaccination. After that, the immunity level slowly decreases in the same way as that of all susceptible individuals with the same immunity level (Goel et al. 2021).

Therefore, we augment the model by adding a compartment, representing newly vaccinated individuals acquiring immunity after vaccination. Similarly as for susceptible and infected individuals, the size of this compartment is counted separately for each immunity level over time, and is denoted by $V(t,\omega )$. The process of acquiring immunity from vaccination (in a relatively short period after vaccination) is modeled in a similar way to the increase of immunity during illness, namely by the equation

4.1 $$\begin{aligned} \dot{\omega }(t) = h(\omega (t)), \quad \omega (\tau ) = \xi , \quad t \ge \theta , \end{aligned}$$

where $\xi \in [0,1]$ is the initial immunity level at the time of vaccination $\tau$. The function $h(\omega ): [0,1] \rightarrow [0,\infty )$, represents how fast immunity builds up at the current immunity level $\omega$. Presumably, it is a decreasing function, with $h(0) > 0$ and $h(1) = 0$, that is, with similar properties as the function *g*.

When reaching their individual maximum immunity level, newly vaccinated individuals leave the vaccinated compartment and are counted as susceptible individuals with the attained new immunity level. This means that their decrease in immunity will change in the same way as for susceptible individuals of the same immune level. The transition process from vaccinated to susceptible occurs with a rate $r(\omega )$, so that $1/r(\omega )$ is the average duration of increase in immunity depending on the current immunity level.

Since vaccinated individuals behave in their activities as susceptible, the infectiousness of the environment, *D*(*t*), takes the form

4.2 $$\begin{aligned} D(t) = \frac{c_I\int _0^1 i(\omega )I(t,\omega ) \mathrm{\,d}\omega }{c_I\int _0^1 I(t,\omega ) \mathrm{\,d}\omega + c \int _0^1 (S(t,\omega ) + V(t,\omega ))\mathrm{\,d}\omega }. \end{aligned}$$

The overall model with vaccination takes the form

4.3 $$\begin{aligned}{} & {} \frac{\partial }{\partial t} S(t, \omega ) + \frac{\partial }{\partial \omega }(f(\omega ) S(t, \omega ) ) = - \left( c D(t) \sigma (\omega ) \right. \left. + v(t,\omega ) \right) S(t, \omega ) + \rho (\omega ) I(t,\omega ) + r(\omega ) V(t,\omega ), \nonumber \\\end{aligned}$$

4.4 $$\begin{aligned}{} & {} \frac{\partial }{\partial t} I(t, \omega ) + \frac{\partial }{\partial \omega }(g(\omega ) I(t, \omega ) ) = cD(t) \sigma (\omega ) (S(t, \omega ) + V(t,\omega )) - (\rho (\omega ) + \mu (\omega )) I(t, \omega ), \end{aligned}$$

4.5 $$\begin{aligned}{} & {} \frac{\partial }{\partial t} V(t, \omega ) + \frac{\partial }{\partial \omega }(h(\omega ) V(t, \omega ) )) = - \left( c D(t) \sigma (\omega ) \right. \left. + r(\omega ) \right) V(t, \omega ) + v(t,\omega ) S(t,\omega ), \end{aligned}$$

with initial conditions

4.6 $$\begin{aligned} S(0,\omega ) = S^0(\omega ), \quad I(0,\omega ) = I^0(\omega ), \quad V(0,\omega ) = 0, \quad \omega \ge 0. \end{aligned}$$

and boundary conditions

4.7 $$\begin{aligned} S(t,1) = 0, \quad I(t, 0) = 0, \quad V(t,0) = 0, \quad t \ge 0. \end{aligned}$$

#### Remark 4.1

As long as the immunity level of people is not measured in reality, the dependence of *v* on $\omega$ is an idealization. The time from last vaccination or from the last infection could be considered (as practiced in reality) as a proxy for the individual immunity level. It is also possible to consider the vaccination rate depending on time only: $v = v(t)$. In this case one can formally substitute $v(t,\omega ) = v(t)$ in the equations, which means equal vaccination rate for all $\omega$.

#### Remark 4.2

Since $V(0,\omega ) = 0$, the formulae for the basic reproduction numbers remain the same as in the no-vaccination case.

### Optimal vaccination policies

In this subsection we use the model involving the vaccination rate $v(t,\omega )$ to formulate an optimal control problem, which reflects the desire of acting in an rational way. Literature considers a number of reasonable objectives for public health interventions, in particular vaccination, involving the burden on hospitals, the number of sick individuals, the cost of policy measures, as well as direct or indirect economic losses due to the disease or due to the policy measures, see e.g. Bloom et al. (2020); Caulkins et al. (2021). We focus on the following three objectives (to be minimized) posed on a fixed time-horizon [0, *T*].

(i) The most important objective is expressed by the total number of deaths; on average this number represents also the total number of sick people, hence it also reflects the economic cost of the epidemics in absence of additional restrictive measures such as partial lock-down (not employed anymore in most of the countries after 2022).

(ii) Due to various reasons (religious, disbelief, feeling of violation of freedom, etc.) a part of the society is not willing to vaccinate; this is the reason because of which in several European countries the vaccination level is rather low. The decision makers, i.e. governments have to take into account the social tension created by compulsory vaccination and the resulting “social disutility”. Consideration of disutility directly resulting from policy measures is not typical in the literature, although it is a factor that often strongly influences the real decision maker (especially at a political level). We refer to Bloom et al. (2020), Section 4.2 (after (4)), where social disutility of policy measures is involved in the objective function.

(iii) The cost of vaccination, which is perhaps less significant than the first two especially in public health emergency situations.

The first objective is clearly contradictory to the other two. Therefore, in the spirit of Pareto’s approach to multi-criteria optimization problems, we define the weighted aggregated objective to be minimized as

4.8 $$\begin{aligned} J(v):= & {} \int _0^T \int _0^1 \mu (\omega )I(t, \omega ) \mathrm{\,d}\omega \mathrm{\,d}t + \alpha \int _0^T \int _0^1 v(t, \omega ) ^2 \mathrm{\,d}\omega \mathrm{\,d}t \nonumber \\{} & {} +\beta \int _0^T \int _0^1 v(t, \omega )S(t, \omega ) \mathrm{\,d}\omega \mathrm{\,d}t \end{aligned}$$

Here, $\alpha \ge 0$ and $\beta \ge 0$ are weighting parameters. The choice of the above quadratic specifications of the disutility function is somewhat arbitrary, although the quadratic form is not needed in the subsequent numerical simulations. This choice reflects the fact that the social disutility marginally increases with the vaccination rate. The optimization (minimization) is subjected to the constraints (4.2)–(4.7) and the control constraint $v(t,\omega ) \ge 0$.

In this paper, we do not deeply investigate issues as existence of an optimal solution, necessary optimality conditions, convergence of numerical methods, etc. However, due to the linear-convex form of the objective functional and the linearity of the equations (4.3)–(4.5) with respect to *v*, one may expect that an optimal solution exists and the optimal control is Lipschitz continuous. Although this is far not enough to claim convergence of our numerical approach, the results of the numerical experiments (see the next section) and the pertaining sensitivity analysis support such a claim.

The numerical approach we employ is the so-called *direct method* in optimal control, which consists of direct discretization of the equations and the objective functional in time and space (for $\omega$), as briefly described in Subsection 5.1.

## Numerical experiments

In the following section we provide several purely illustrative numerical experiments for the evolution of the model dynamics with and without vaccination. Moreover, we also analyze the impact of optimal vaccination policies among sub-populations with differing immunity level.

### Numerical approximation

In order to obtain numerical solution to (4.2)–(4.7) we use the so called upwind scheme which is of first order accuracy, see LeVeque 2002. Consider for example equation (4.3), which can be written in the following way:

5.1 $$\begin{aligned} \frac{\partial }{\partial t}S(t, \omega ) + \frac{\partial }{\partial \omega }\big (f(\omega ) S(t,\omega )\big ) = F^S\big (t, \omega , D(t), Z(t,\omega )), \end{aligned}$$

with the initial/boundary conditions (4.6) and (4.7). Here, $Z(t,\omega ):= \big (S(t,\omega ), I(t,\omega ), V(t,\omega ) \big )$ and $F^S$ is the right-hand side of (4.3).

In order to describe the numerical scheme, we define the uniform mesh $\omega _j, j=1,...,M+1$, in the $\omega$-dimension with step size $\Delta \omega = \omega _{i+1} - \omega _{i}$. Similarly, in the *t*-dimension we define the mesh $t_i, i=1,...,N+1$, with step size $\Delta t = t_{i+1} - t_{i}$. The upwind scheme is represented by the following implicit recurrent equation:

$$\begin{aligned} \begin{aligned} \frac{S(t_{i+1},\omega _j) - S(t_{i},\omega _j)}{\Delta t}&= - f(\omega _j)\Big (\frac{ S(t_{i},\omega _{j+1}) - S(t_{i},\omega _{j})}{\Delta \omega }\Big ) \\&\quad + F^S(t_{i},\omega _{j},D(t_i), Z(t_{i},\omega _{j})), \end{aligned} \end{aligned}$$

for $i=1,...,N$ and $j=1,...,M$. From the boundary condition (4.7) we have that $S(t_{i},\omega _{M+1})=0$, for every grid point $t_i$.

The scheme has to take into consideration also the sign of the functions *f*, *g* and *h*. For the equations (4.4)–(4.6) we have to change *f* with *g* or *h*, the numerator on the right-hand side to $I(t_{i},\omega _{j}) - I(t_{i},\omega _{j-1})$ or $V(t_{i},\omega _{j}) - V(t_{i},\omega _{j-1})$, and account for the zero boundary condition (4.7).

A necessary condition for convergence of the upwind scheme is the Courant- Friedrichs-Lewy condition (CFL), see Courant (1967). In our case this condition takes the form $\frac{u \Delta t }{\Delta \omega } \le C < 1$, where $u = \max _\omega \{f(\omega ), g(\omega ), h(\omega )\}$ and *C* is the Courant number for the problem. We tested the scheme with various step sizes that satisfy the CFL condition in order to obtain reliable numerical results.

### Model parameters – the baseline scenario

We outline the parameters selected for a baseline case, which will be used in the subsequent numerical analysis. In addition, the base case values will be varied to study the solution sensitivity. It is important to note that all values are selected for illustrative purposes only and do not correspond to a specific disease: while the current work presents a general model and analyzes some of its properties, substantial empirical work remains to be done in order to apply it to real-world data.

The initial distribution of the compartment sizes is chosen consistently with the boundary conditions provided in (2.7), respectively (4.7). For the distribution of the susceptible population at time zero, we take a linear function $S^0(\omega ) = 1.9 (1-\omega )$, so that 95% of the population is susceptible at time $t = 0$. A parabola is chosen for modeling the distribution of the initial infected population, $I^0(\omega ) = 0.3 \,\omega (1-\omega )$, which gives initial infected population $5 \%$ of the total. There are no vaccinated individuals at the beginning: $V^0(\omega ) = 0$.

The parameters and functions for modeling contact rates, infectiousness, recovery and mortality are summarized in Table 1.

**Table 1:** Parameters and functions used in the numerical examples, $\omega \in [0,1]$

Model parameters and functions		
Contact rate of susceptible individuals	*c*	8
Contact rate of infected individuals	$c_I$	3
Susceptibility	$\sigma (\omega )$	$(1.5-\omega )^3$
Recovery	$\rho (\omega )$, $r(\omega )$	$\frac{120^{\omega ^{1.3}}}{40}$
Infectiousness	$i(\omega )$	$0.3(1 - 0.9\omega )$
Mortality	$\mu (\omega )$	$0.01(1 -\omega )$
Immunity decrease (susceptibles)	$f(\omega )$	$-0.005\omega$
Immunity increase (infected individuals)	$g(\omega )$	$0.04(1-\omega )$
Immunity increase (vaccinated individuals)	$h(\omega )$	$0.02(1-\omega )$
Disutility cost parameter	$\alpha$	0.00001
Administration cost parameter	$\beta$	0.0005

Table 1 also shows the specific functions *f*, *g* and *h*, which are specified as affine, such that the sign of their slope determines gain or loss of immunity over time.

In order to propose reasonable functional specifications, the following factors were taken into account. Susceptibility, infectiousness and mortality, $\sigma , \, \iota$, and $\mu$, decrease when immunity is higher. The specific form of the recovery rates $\rho$ and *r* ensures that the average duration of recovery is shorter when immunity is higher; for zero immunity $\rho (0) = 1/40$, thus for $\omega = 0$ the average duration of recovery is 40 days; for $\omega = 0.5$ the average duration is about 6 days, for $\omega = 1$ it is negligible.

Figure 1 shows the resulting characteristic curves from solving (2.2) and (2.1).

### Numerical results without vaccination

We start with numerical simulations of the baseline model, excluding vaccination.

**Fig. 2:**
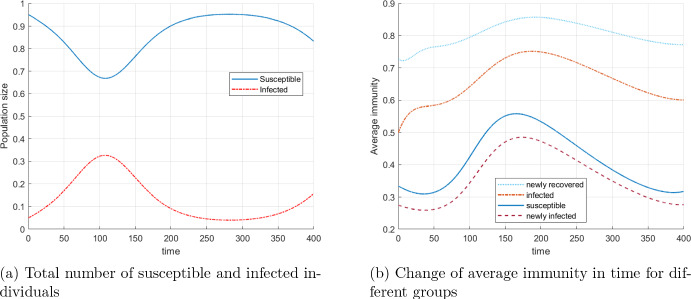
Evolution of epidemiological population groups without vaccination

Figure 2 gives an overview of the development of the epidemic over the simulation horizon with two graphs. The first wave of the epidemic plus the emergence of a second wave is shown in Fig. 2a. Here, the numbers of individuals in the compartments of susceptible and infected individuals are aggregated over all immunity levels $\omega$. Figure 2b captures the development of the average immunity level for susceptible and infected individuals, i.e. $\int _0^1 \omega S(t,\omega ) \mathrm{\,d}\omega / \int _0^1\,S(t,\omega ) \mathrm{\,d}\omega$, etc. In addition, the average immunity level of newly infected and newly recovered individuals is illustrated. In particular, one can observe an increase of the average immunity level in all compartments after the number of infected individuals peaks (compare with the first plot 2b). In the susceptible compartment the average immunity increases due to the inflow of recovered individuals with high immunity level, until the number of infected individuals becomes low enough. The magnitude of the immunity increase for a given initial immunity level mainly depends on the choice of the function *g*.

**Fig. 3:**
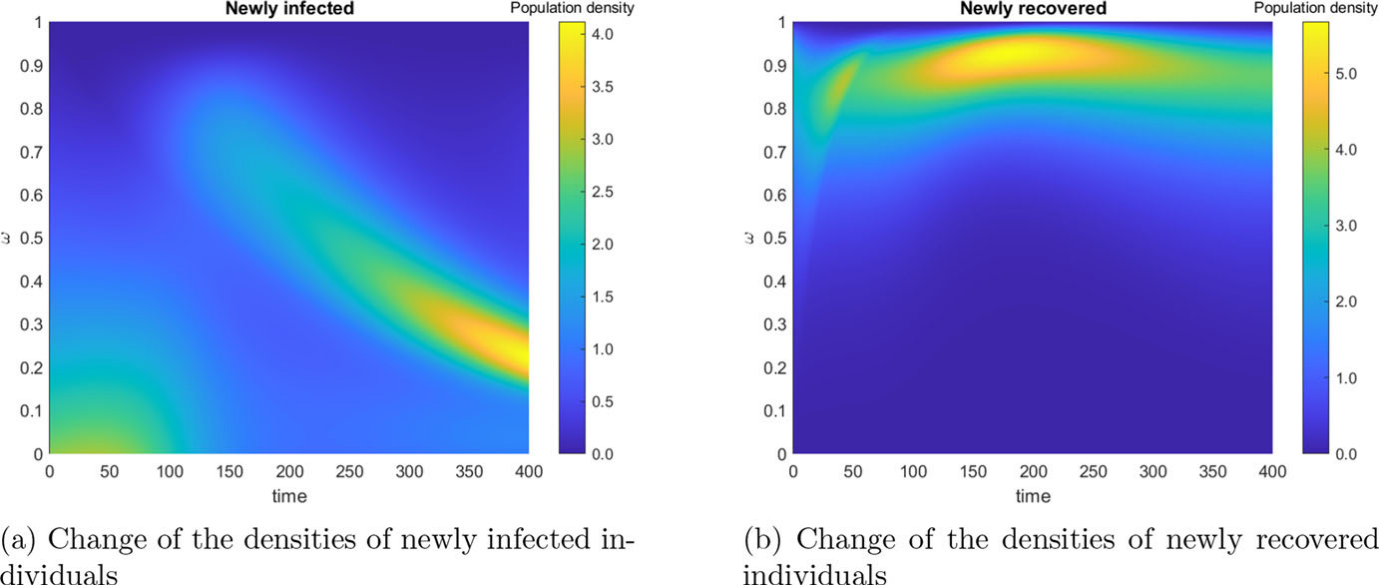
Evolution of the normalized densities of newly infected and newly recovered individuals

Figure 3 presents the evolution of the densities of the newly infected individuals and the newly recovered individuals. For each time *t* (on the horizontal axis), the related vertical cut pictures the normalized densities $\sigma (\cdot )S(t,\cdot )/ \int _0^1 \sigma (\omega ) S(t,\omega ) \mathrm{\,d}\omega$ and $\rho (\cdot ) I(t,\cdot )/ \int _0^1 \rho (\omega ) I(t,\omega ) \mathrm{\,d}\omega$. The bright yellow spots correspond to higher densities and the dark blue color corresponds to a low density. The immunity of the newly recovered individuals is much higher than that of the newly infected, which is to be expected, given that the immunity level increases during the infection.

**Fig. 4:**
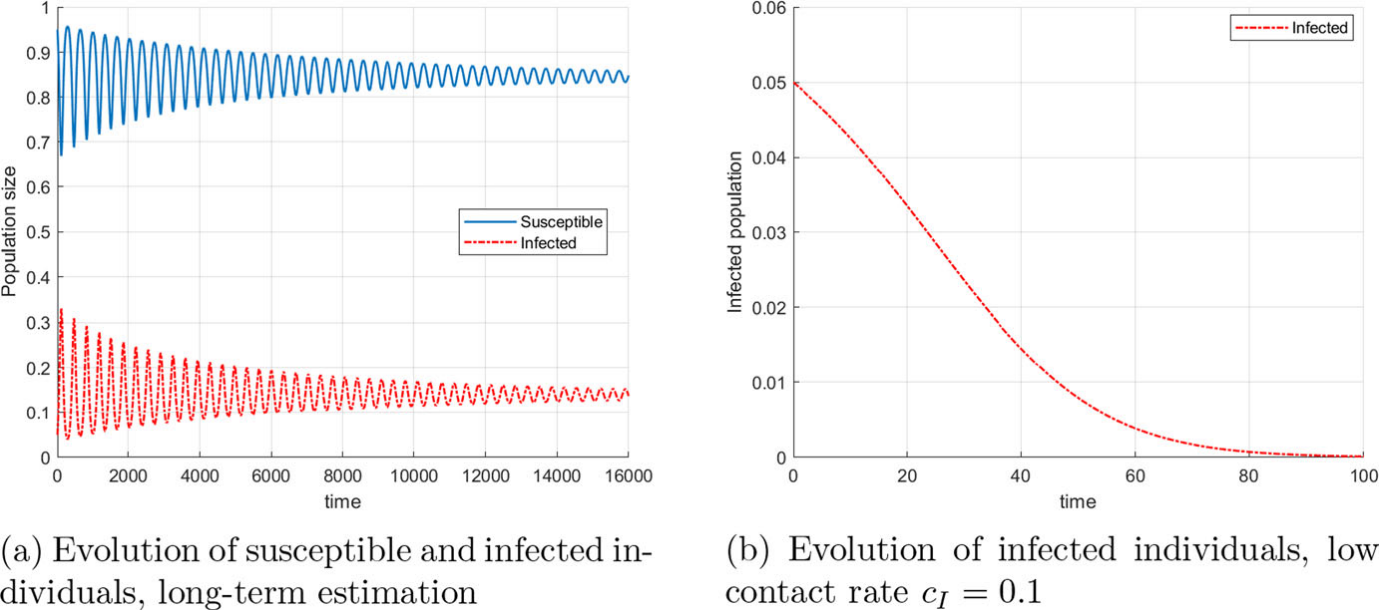
Evolution of epidemiological groups with no mortality rate

In order to show the long term behavior of the model, we simulate the baseline scenario without mortality, $\mu (\omega )=0$, on a longer period of time, $T= 16000$ days. As depicted in Fig. 4a, the system exhibits an oscillatory behaviour with declining amplitude, which suggests convergence towards an endemic equilibrium. Figure 4b shows the decrease of the group of infected individuals when the contact rate $c_I$ of infected individuals has the lower value 0.1. For this value the condition in Proposition 3.3 is satisfied and we observe convergence to an disease-free equilibrium.

### Simulations with constant vaccination

Before considering the vaccination rate $v(t,\omega )$ as a control variable, we analyze in the current subsection the effect of a constant and $\omega$-independent vaccination rate, $v(t,\omega ) \equiv v$, on the evolution of the epidemics in the baseline case.

**Fig. 5:**
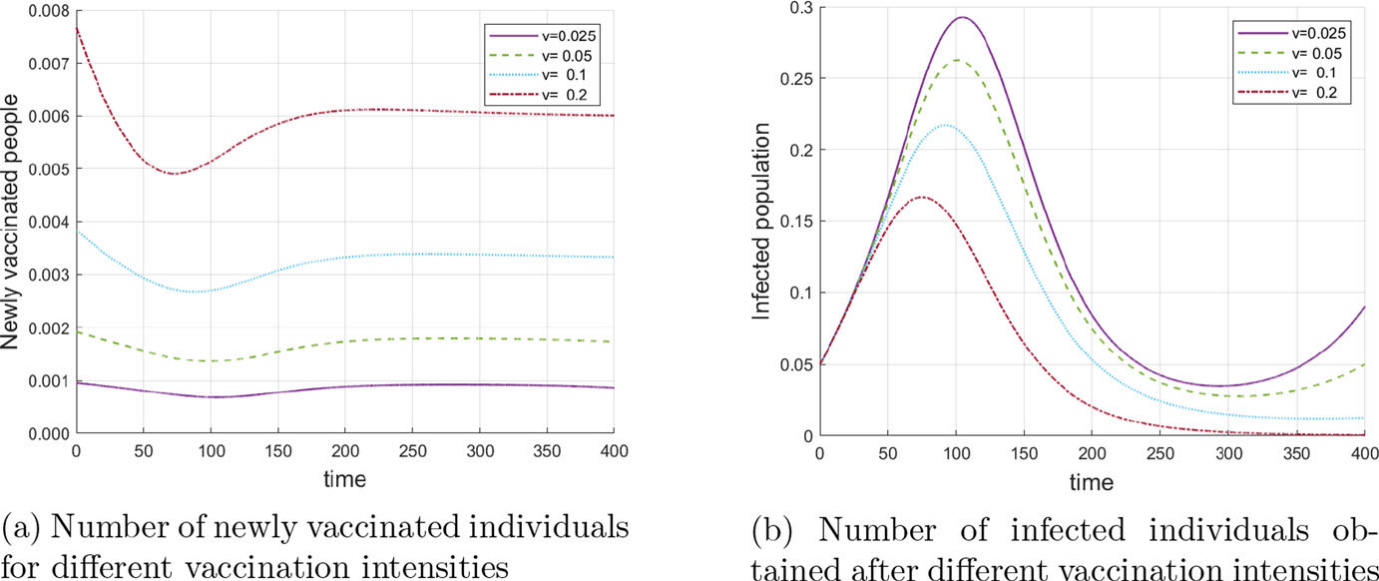
Evolution of epidemiological population groups with constant vaccination

In Fig. 5a one can see the number of newly vaccinated individuals, $v \int _0^1 S(t,\omega ) \mathrm{\,d}\omega$. Figure 5b presents the number of infected individuals for various vaccination rates *v*. Observe that for $v(t,\omega )=0.2$ the number of infected individuals approaches zero after the first wave.

**Fig. 6:**
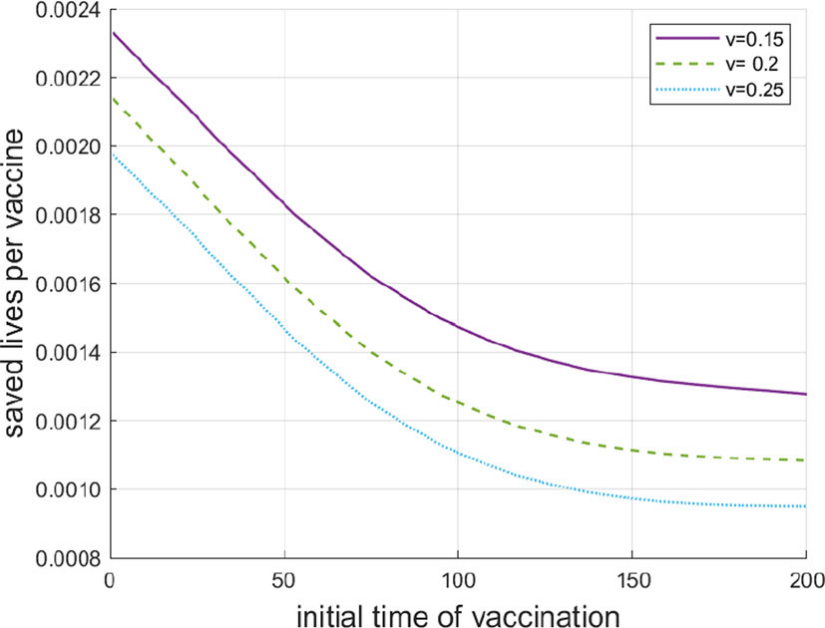
Saved lives per vaccine depending on the initial date of implementing vaccination and the vaccination rate *v*

In the next experiment we consider the results of vaccination depending on the initial time of implementation of the vaccine. This is done for three constant vaccination rates: $v = 0.15, \;0,20, 0.25$. Figure 6 shows the saved lives per vaccination (compared with the case without vaccination) depending the on initial time. It is remarkable that the three curves are strictly convex, which means that a delay in the implementation of a vaccine causes more deaths in earlier stages of the epidemics than in later stages. One reason for that is the increase of the average immunity level in the course of epidemics due to infections (manifestation of the herd immunity in the present model) and the marginal increase of immunity due to vaccination is smaller.

### Optimal vaccination

Now, we analyze numerically the control problem, formulated in Subsection 4.2. The control function *v* is considered as piece-wise constant. We use the direct method of optimal control and direct discretization of equations (4.3)–(4.5) by the scheme described in Sect. 5.1. Furthermore, the integral in (4.8) is approximated by a second-order quadrature formula. For solving the resulting mathematical programming problem we utilize the SQP method available as a function of the MATLAB™ Optimization Toolbox. The parameters of the baseline scenario are shown in Table 1.

**Fig. 7:**
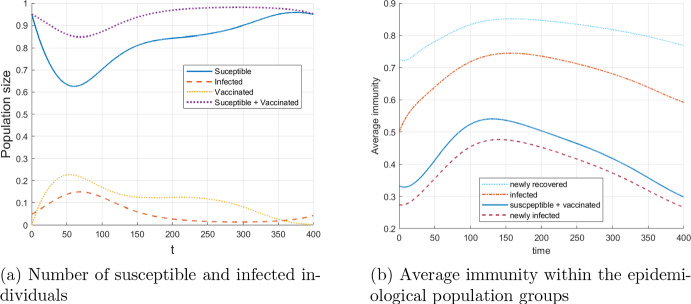
Evolution of epidemiological population groups with optimal vaccination applied

Figure 7 shows the effect of optimal vaccination in the baseline case. In addition to the overall numbers of susceptible and infected individuals, the yellow line in Fig. 7a indicates the total number of individuals in the vaccinated group. These are individuals who are in the process of acquiring immunity due to the vaccination. As expected, the overall level of infected individuals is greatly reduced compared to the baseline case without vaccination in Fig. 2a. Surprisingly, the reason for that is not that the average immunity of the non-infected population is standingly higher than that of the non-vaccinated population. In Fig. 7b we see the average immunity of all compartments. Comparing the blue line on the previous plot 2b with that on 7b we see that they are quite similar, with the difference that the average immunity of the vaccinated population increases to its maximum substantially earlier than that of the non-vaccinated population. Thus the immunity level of the infected population is much higher exactly in the expansion face of the epidemics which leads to less infections. Later on, the immunity level of the non-infected population catches up due to the higher herd immunity.

**Fig. 8:**
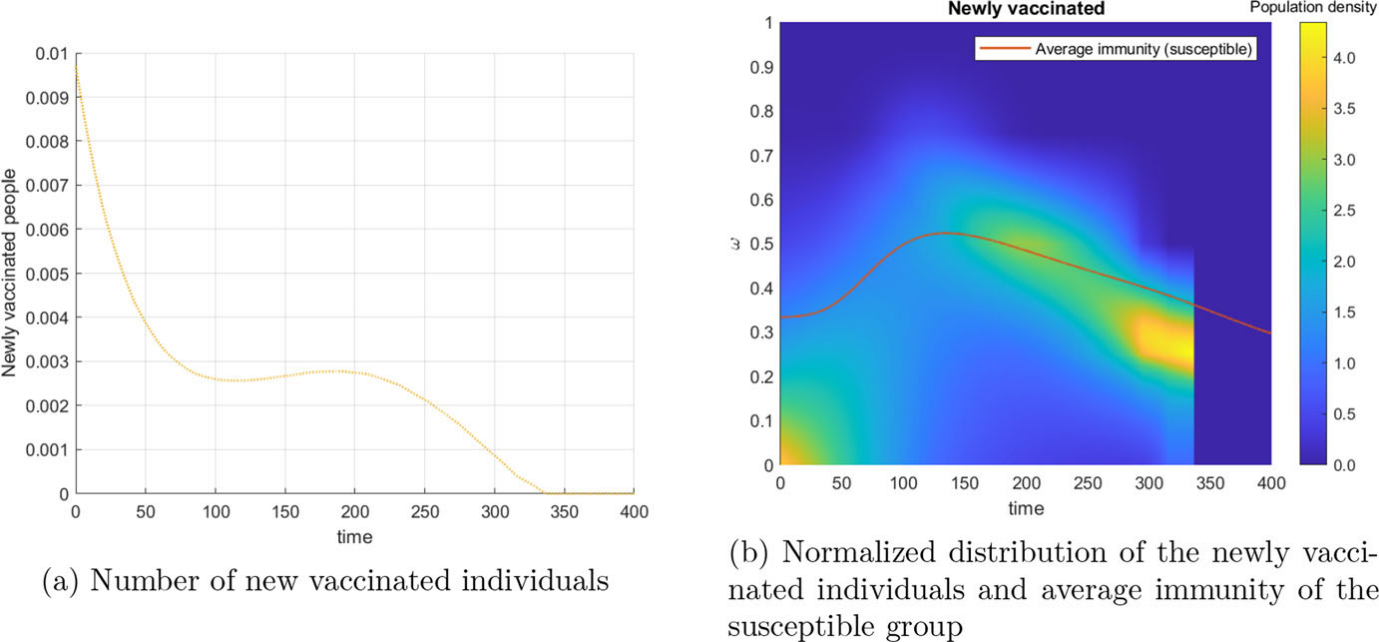
Optimal Strategy for the baseline scenario: Administered vaccines, i.e. newly vaccinated individuals and comparison with the average immunity level of the susceptible group

The optimal vaccination policy is analyzed in Fig. 8. Figure 8a shows the optimal total number of newly vaccinated individuals. It can be seen that the main effort should be concentrated immediately at the beginning in order to boost immunity in the population, while only a smaller effort is dedicated later-on in order to replace the waning immunity and to maintain a low level of infections. Due to the finite horizon of the optimal control, the vaccination is terminates before the end of the simulation period.

Because we allow vaccination efforts to depend on the immunity level, optimal vaccination policy is not only a matter of the overall level and timing of vaccination. Figure 8b shows the distribution of the application of vaccines to individuals with differing immunity level over time. Again, the abrupt change at the end of the horizon is due to the stop of vaccination. It can be seen that, at the beginning – when the overall effort is high, vaccination tends to be given to individuals with lower immunity level than the average immunity level in the susceptible group. The levels of immunity of vaccinated individuals then catch up with the average immunity level of susceptible individuals after around 150 days, and then follow the general decrease of the average level of immunity.

**Dependence on the time-horizon and model predictive control.** In practice, any vaccination policy has to be revised after some time to catch up with new information. In particular, the improved medical understanding of the disease, changes in the death rates, new variants of the pathogens may emerge and enhanced vaccines may be developed. In terms of control, a new optimization is done after some time with updated information, which is known as Model Predictive Control in the literature. The question arise, whether our model is suitable for such revisions.

In order to apply Model Predictive Control, we may solve the optimization problem on a relatively short horizon, e.g. $t=400$ days in the baseline case, apply the obtained solution during an even shorter time horizon, say 70 days, then update the model parameters and the current real state of the epidemic, solve the problem on the next 400 days horizon, and so on.

**Fig. 9:**
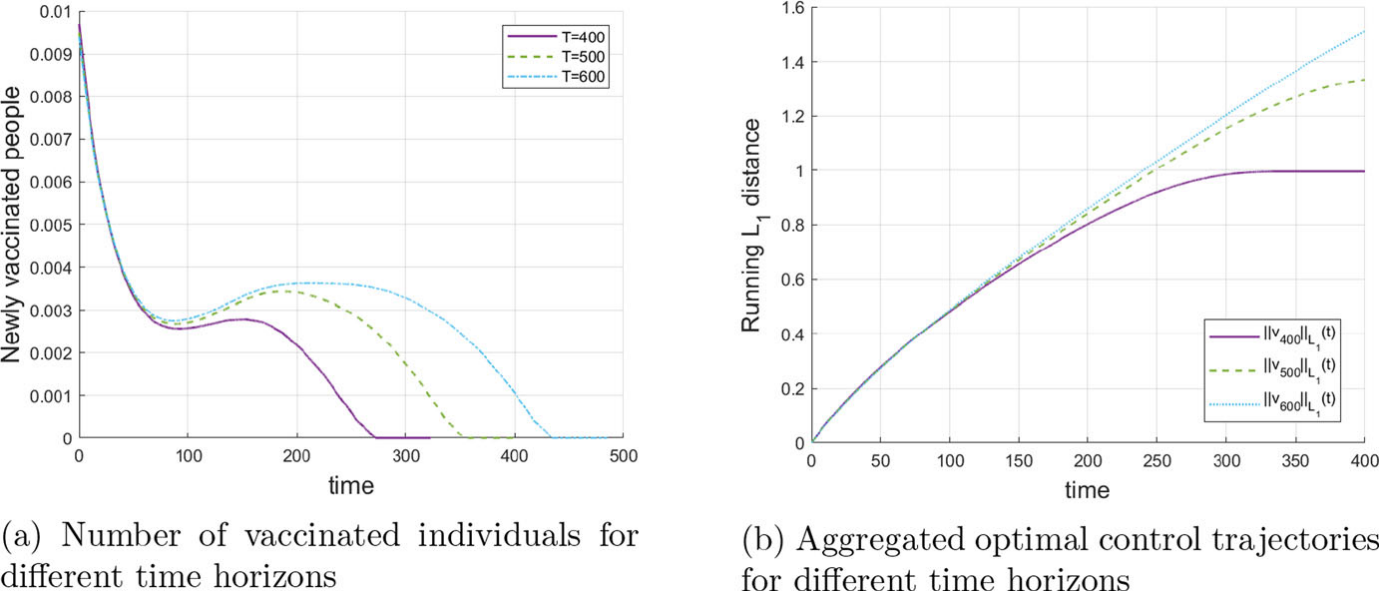
Dependence of the optimal vaccination policy on the time horizon [0, *t*], $t = 400, 500, 600$

Such an approach only works well if the results with different planning horizons do not vary too much over the shorter time period (here 70 days). This is tested in Fig. 9, which shows the dependence of the optimal vaccination policy on the chosen time horizon [0, *t*] on which the optimization problem is solved. The plot on the left indicates that the total number of optimally vaccinated people is practically independent of the time horizon over the first 70 to 100 days. More relevant, the same applies (even on a longer horizon) to the aggregated (in $\omega$) vaccination effort (the right Fig. 9). So, it seems to be reasonable to apply Model Predictive Control.

**Trade-off between the objectives.** The objective (4.8) puts together three objectives: the number of deaths and two kinds of vaccination costs. All these goals are relevant for decision-making in public health, but also are contradictory. In order to formulate the overall objective, weights are applied, modeling the relative importance of the individual objectives.

Although the analysis of the optimization problem so far has been focused on the analysis of one ("standard") choice of these weights (see the baseline scenario defined in Table 1), it is possible to go deeper by analyzing the efficient frontier (or Pareto frontier). With conflicting goals, vaccination strategies can be compared by showing the vectors of their respective partial objectives (deaths, number of vaccinations, social cost) in a plot. Iterating over the possible weight combinations ($\alpha , \beta$) and plotting the values of partial goals, one gets the Pareto frontier. From the viewpoint of a decision maker, the efficient frontier depicts those combinations of conflicting goals that are achievable at the best.

Having three objectives, the efficient frontier is a 2-dimensional manifold in the 3-dimensional space. In order to show a two-dimensional picture, we vary only the weight of the administration cost, $\beta$, and plot the Pareto curve, holding fixed the parameter $\alpha$ as in the baseline case (the weight of the deaths is fixed to 1 by normalization of the overall objective function). Varying the weight $\beta$ in the range from 0.001 to 0.02 and calculating the corresponding optimal vaccination policy, we obtain (a part of) the efficient Pareto curve.

We consider three cases for the vaccination control *v*: (i) the control may depend on time and immunity level as in the previous considerations, that is, $v = v(t,\omega )$; (ii) the control depends only on time, $v = v(t)$, and the vaccination is uniformly distributed over individuals of different $\omega$; (iii) the control is constant across time and immunity levels. The first case represents an idealized situation in which full information about the immunity level is required, while in the other two scenarios such information is not needed. This setup allows to quantify the effects of available information on the objective values, similar to the concept of the value of information in stochastic optimization.

**Fig. 10:**
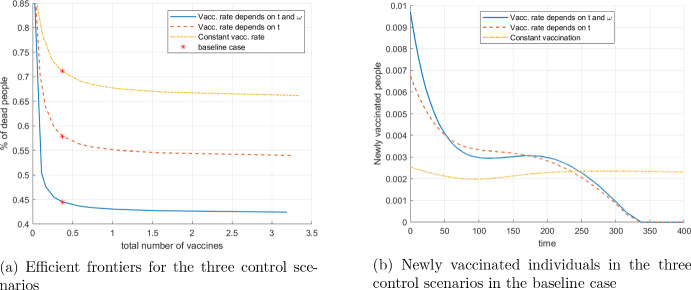
The left plot presents the efficient frontier for the three control scenarios; the red dots on the Pareto curves indicate the Pareto points corresponding to the baseline case. The right plot depicts the time-dependence of the number of newly vaccinated individuals in the three control scenarios in the baseline case

Fig. 10a shows the efficient frontiers for the three scenarios, and also depicts the locations of the optimized baseline scenarios ($\beta$ is chosen as in the baseline scenario). Summarizing, the blue curve in Fig. 10a represents the optimal vaccination administration cost (in term of the number of vaccinations) versus the optimal achievable total percentage of deaths for case (i). For any point (*b*, *d*) on this efficient frontier, *d* is the minimal percentage of deaths that can be achieved by vaccination budget *b*. Vice versa, if it is intended to limit the number of deaths to *d*%, then at least *b* are the necessary vaccination costs. By the strict convexity of the Pareto curve, the less is the number of deaths, the more costly it becomes to obtain any additional reduction of the of deaths. Similar explanation applies to the other two control scenarios. Note that the baseline scenario with no vaccination at all is efficient for all three cases. It lies on the point, where all three curves touch the ordinate.

Fig. 10b shows the overall control strategy (aggregated over $\omega$ in case (i)) for the three baseline cases, related to the red points in Fig. 10a. It suggests that, compared to the other two scenarios, more people should be vaccinated at the beginning of the epidemics, if information on the distribution of $\omega$ is available (control scenario (i)). The value of information (and of capacity to act) is demonstrated by the mutual positioning of the three curves in Fig. 10a: the Pareto frontiers of cases with more information or capacity to act, clearly dominate the other curves.

### Comparative analysis

**Fig. 11:**
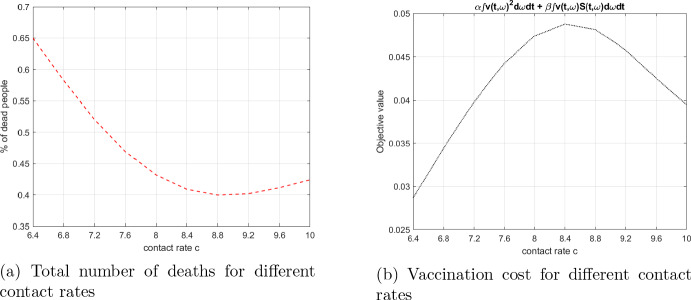
Objective function components for different contact rates

We stay with the baseline case and the optimal control problem, considered so far, and analyze the effect of parameter changes on the objective value. As contact rate plays a significant role in pathogen transmission, we vary the baseline values for $c=8$ and $c_I=3$, by multiplying these parameters by a factor ranging from 0.8 to 1.25. For each parameter value we calculate the optimal vaccination policy and plot the corresponding optimal percent of deaths in Fig. 11a and the remaining part of the optimal objective value in (4.8) (representing the disutility from, and cost of vaccination) in Fig. 11b. In both plots, the x-axis shows the values of the contact rate *c*.

In the graph in 11a the lowest mortality cost results from the contact rate $c=8.8$ and in the second plot 11b the highest vaccination cost occurs for $c=8.4$. One can observe that a lower contact rate does not necessarily imply lower mortality. When the contact rate increases, the optimal vaccination efforts also increase, but only up to a point. The vaccination leads to lower mortality rates, but we can also observe from figure 11b, that the vaccination costs for highest values of the contact rate is reduced. This fact can be explained by the effect of herd immunity. With higher contact rate more people obtain immunity from infection and the effect of vaccination is relatively smaller. In this sense, the vaccination and the herd immunity appear as substitutes when the contact rates are sufficiently high.

## Discussion

In this study, we introduce and analyze an epidemiological model that explicitly incorporates the impact of waning immunity following infection or vaccination. The model differs substantially from previous approaches in the epidemiological literature (White and Medley 1998; Rouderfer and Becker 1994; Barbarosa and Röst 2015; Ehrhardt et al. 2019). It consists of a system of two PDEs of first order. When vaccination is considered, a third equation is added. The complexity of the model lies in its mathematical intricacies, primarily the inclusion of a nonlocal term, which necessitates the integration of state variables in a nonlinear fashion. This complexity is further amplified by the fact that the velocity fields are different in different equations.

A qualitative study of the model is provided that includes existence of a global solution, conditions for decay of the epidemics from a given state are obtained, and basic reproduction numbers under various information patterns.

In a further step, we introduce vaccination strategies and formulate an optimal control problem with three objectives: the total number of deaths, the social discomfort created by the pressure that people experience when the vaccination effort is high, and the direct costs of vaccination. Using plausible scenarios of vaccination, numerical results provide insights into the dynamics of the epidemiological populations involved, including the waning immunity with and without vaccination. With respect to the optimal vaccination strategy the model provides insights into the influence of different factors on the optimal policy and performance. An interesting fact, for example, is that vaccination efforts and herd immunity act in a certain sense as substitutes: above a threshold value of the contact rate, further increase of the contact rate leads to lower vaccination rate. Below this threshold value, increase of the contact rate leads to increase of vaccination rate. In addition, we determine the efficient frontier between vaccine administration costs (direct and indirect) and the number of deaths, using three different control settings: optimal control policy which is independent of time and immune level, optimal policy that depends only on the time, and optimal policy depending on time and immunity level.

Although the model has some striking features such as the description and coupling of the elicitation of immune responses to the epidemiological process it can only be considered as a first step towards a more detailed description of immune responses and their waning over time. In particular, the model covers the immune response in a broad sense, without differentiating between innate and adaptive responses and their antibody and cellular branches. It does not account for their unique dynamical attributes, such as the time lag between the two, and their varying intensities contingent on the infectious disease under investigation. There is still substantial clinical and epidemiological empirical work to be done to this type of model with relevant immuno-epidemiological data. Despite its limitations, the model demonstrates that a mathematical representation of these dynamic processes is feasible. This could pave the way for a deeper comprehension of the processes in question and the assessment of related interventions.

## Appendix: Proof of Theorem 3.1

Since the horizon *T* may change in the subsequent considerations, at some places we use the notation $\Gamma ^T:= [0,T] \times [0,1]$. The space of all continuous functions from a set $X \subset \mathbb {R}^n$ to $\mathbb {R}$ is denoted by *C*(*X*), with the usual norm denoted by $\Vert \cdot \Vert _{C(X)}$. For $\varphi = (\varphi _1,\ldots ,\varphi _k): X \rightarrow \mathbb {R}^k$ denote $\Vert \varphi \Vert _{C(X)}:= \sum _{i=1}^k \Vert \varphi _i \Vert _{C(X)}$. The spaces $L^1(0,T)$ and $L^\infty (0,T)$ are defined as usual. Further, $\mathcal{L}(X) \subset C(X)$ is the subspace of all Lipschitz continuous functions with Lip(*x*) denoting the (minimal) Lipschitz constant of $x \in \mathcal{L}(X)$. We abbreviate

$$\begin{aligned} F = \left( \begin{array}{c} F^S \\ F^{I} \end{array} \right) \qquad Z = \left( \begin{array}{c} S \\ I \end{array} \right) \quad \text{ or } \; Z = (S,I), \quad \text{ etc. } \end{aligned}$$

The existence theorem presented below will be formulated in the terms of a general function *F* in the equations (3.7), (3.8), and with a general relation between the functions *D* and (*S*, *I*). Namely, instead of equation (2.3) we set

6.1 $$\begin{aligned} D = \mathcal{D}(Z), \quad \mathcal{D}: \textrm{dom}\,\mathcal{D}:= \{ Z \in \mathcal{L}(\Gamma ^T): \, Z \ge 0\} \rightarrow L^1(0,T), \end{aligned}$$

where $\mathcal{D}$ has the following properties: there exists constants $a > 1$ and $L_\mathcal{D}$ such that

6.2 $$\begin{aligned}{} & {} 0 \le \mathcal{D}(Z)(t) \le a-1, \end{aligned}$$

6.3 $$\begin{aligned}{} & {} | \mathcal{D}(Z_1)(t) - \mathcal{D}(Z_2)(t) | \le L_\mathcal{D}\, \max _{\omega \in [0,1]} | Z_1(t,\omega ) - Z_2(t,\omega )|, \nonumber \\{} & {} \quad \text{ for } \text{ a.e. } t \in [0,T], \;\; Z, Z_1,Z_2 \in \textrm{dom}\,\mathcal{D}. \end{aligned}$$

Keeping in mind the specific form of the functions $F^S$ and $F^{I}$ in (3.1), (3.2), we assume in the general case that there exist constants *M* and *L* such that

6.4 $$\begin{aligned}{} & {} \!\!\! |F(t,\omega ,d,s,i)| \le Ma(|s| + |i|), \end{aligned}$$

6.5 $$\begin{aligned}{} & {} \!\!\! |F(t,\omega ,d,s,i)| - F(t_1,\omega _1,d_1,s_1,i_1)| \le La \big ( (|s| + |i| ) (|t-t_1| \nonumber \\{} & {} \quad + |\omega - \omega _1| + |d-d_1|)+ |s-s_1| + |i-i_1|\big ) \end{aligned}$$

for all $t, t_1 \in [0,1]$, $\omega , \omega _1 \in [0,1]$, $d,d_1,s, s_1,i, i_1 \in \mathbb {R}$ with $|d|, \, |d_1| \le a-1$. Moreover, the following property is fulfilled: for any $d \ge 0$, $(t,\omega ) \in \Gamma$

6.6 $$\begin{aligned} F^S(t,\omega ,d,0,i) \ge 0 \;\;\; \forall i \ge 0, \qquad F^I(t,\omega ,d,s,0) \ge 0 \;\;\; \forall s \ge 0. \end{aligned}$$

### Theorem 6.1

Let the functions $f, g, S^0,I^0$ satisfy the Standing Assumptions (at the beginning of Subsection 3.1). Let, in addition, the conditions (6.2)–(6.6) be fulfilled. Then there exists $T > 0$, independent of the particular initial data $(S^0, I^0)$, such that the system (3.7), (3.8), (6.1) has a unique nonnegative Lipschitz continuous solution $Z = (S,I)$ on $\Gamma ^T$, satisfying the inequality $\mathcal{D}(Z) \le a-1$.

### Proof

**1.** We begin with some preliminary facts and notations. Due to the properties of *f* and *g*, the functions $(\gamma ,s) \rightarrow \omega ^f[\gamma ](s)$ and $(\gamma ,s) \rightarrow \omega ^g[\gamma ](s)$ are continuously differentiable on a neighborhood of $\Gamma ^T \times [0,T]$ ($T > 0$ is arbitrary here). Denote by $\lambda _\omega$ a common Lipschitz constant of these functions on $\Gamma ^1 \times [0,1]$. Moreover, the functions $\Gamma ^1 \ni \gamma \rightarrow \gamma ^f(\gamma )$ and $\Gamma ^1 \ni \gamma \rightarrow \gamma ^g(\gamma )$ are Lipschitz continuous and we denote by $\lambda _\gamma$ a common Lipschitz constant.

Let us fix the number $T > 0$ such that

6.7 $$\begin{aligned} T \le \min \left\{ 1, \frac{1}{4L\lambda _\omega \, a}, \, \frac{1}{2 La } \right\} . \end{aligned}$$

Notice that *T* does not depend on the initial data $(S^0, I^0)$.

Let us fix a $D \in L^\infty (0,1)$ with $\Vert D \Vert _{L^\infty } + 1 \le a$. Set

6.8 $$\begin{aligned} \lambda := \max \big \{4 \lambda _\gamma , \, 8 a e^{2Ma} (2M(1+\lambda _\gamma ) + L \lambda _\omega ) \big \} \end{aligned}$$

and define the set

6.9 $$\begin{aligned}{} & {} \mathcal{K}_{T,a} := \Big \{ Z = (S,I) \in \mathcal{L}(\Gamma ^T): \, \text{ Lip }(Z) \le (\Vert Z^0\Vert _{C(0,1)} + \textrm{Lip}(Z^0)) \lambda ,\nonumber \\{} & {} \quad \Vert Z\Vert _{C(\Gamma ^t)} \le 2e^{2Ma t} \Vert Z^0\Vert _{C(0,1)} \;\, \forall t \in [0,T], (2.6)\, \text{ and } \, (2.7)\, \text{ are } \text{ satisfied } \Big \}. \nonumber \\ \end{aligned}$$

On $\mathcal{K}_{T,a}$ we define the mapping $\mathcal{F}_{[D]}$ as

6.10 $$\begin{aligned} \mathcal{F}^S_{[D]}(Z)(\gamma ):= & {} \int _{\tau ^f(\gamma )}^t F^S(s,\omega ^f[\gamma ](s), D(s), Z(s,\omega ^f[\gamma ](s))) \mathrm{\,d}s + {\bar{S}}^0(\gamma ^f(\gamma )), \nonumber \\ \end{aligned}$$

6.11 $$\begin{aligned} \mathcal{F}^{I}_{[D]}(Z)(\gamma ):= & {} \int _{\tau ^g(\gamma )}^t F^{I}(s,\omega ^g[\gamma ](s), D(s), Z(s,\omega ^g[\gamma ](s))) \mathrm{\,d}s + {\bar{I}}^0(\gamma ^g(\gamma )), \nonumber \\ \end{aligned}$$

where $\gamma = (t,\omega ) \in \Gamma ^T$. In the next three parts of the proof we shall prove that $\mathcal{K}_{T,a}$ is a nonempty complete metric space, $\mathcal{F}_{[D]}$ maps $\mathcal{K}_{T,a}$ to $\mathcal{K}_{T,a}$, and $\mathcal{F}_{[D]}$ is contractive.

**2.** Let us prove that $\mathcal{K}_{T,a}$ is not empty. For $\gamma \in \Gamma ^T$ we set $S^\#(\gamma ):= {\bar{S}}^0(\gamma ^f(\gamma ))$ and $I^\#(\gamma ):= \bar{I}^0(\gamma ^g(\gamma ))$ (representing the evolution of the initial/boundary data if $F \equiv 0$). Since $Z^0 \in \mathcal{L}(0,1)$ and $S^0(1) = I^0(0) = 0$, the function ${\bar{S}}^0$ is Lipschitz continuous with Lip$({\bar{S}}^0) = \textrm{Lip}(S^0)$. Thus $\textrm{Lip}(S^\#) \le \textrm{Lip}(S^0) \textrm{Lip}(\gamma ^f) \le \textrm{Lip}(Z^0) \lambda _\gamma$. The same applies to $I^\#$, thus the first inequality in the definition of $\mathcal{K}_{T,a}$ is fulfilled by $Z^\#$. The second inequality is also fulfilled since $\Vert {{\mathcal {Z}}}^\#\Vert _C \le \Vert Z^0\Vert _{C}$. The conditions (2.6) and (2.7) are apparently also fulfilled, thus $\mathcal{K}_{T,a} \not =\emptyset$.

Due to the uniform Lipschitz property in the definition of the set $\mathcal{K}_{T,a}$, it is a complete metric space in the metric induced by the norm in $C(\Gamma ^T)$.

**3.** (Invariance of $\mathcal{K}_{T,a}$.) Obviously for $\gamma = (0,\xi )$ we have $\mathcal{F}^S_{[D]}(Z)(\gamma ) = {\bar{S}}^0(\gamma ^f(0,\xi )) = S^0(\xi )$, and for $\gamma = (\tau ,1)$ we have $\mathcal{F}^S_{[D]}(Z)(\gamma ) = \bar{S}^0(\gamma ^g(\tau ,1)) = {\bar{S}}^0(\tau ,1) = 0$, thus $\mathcal{F}^S_{[D]}(Z)$ satisfies the side conditions in (2.6) and (2.7). The same applies to $\mathcal{F}^I_{[D]}(Z)$.

Fix an arbitrary $Z \in \mathcal{K}_{T,a}$. Using (6.4), we have for any $\gamma = (t,\omega ) \in \Gamma ^T$

$$\begin{aligned} | \mathcal{F}^S_{[D]}(Z)(\gamma )|\le & {} |{\bar{S}}^0(\gamma ^f(\gamma ))| + \int _{\tau ^f(\gamma )}^t M a (|S(\theta ,\omega ^f[\gamma ](\theta ))| + I(\theta ,\omega ^f[\gamma ](\theta ))|) \mathrm{\,d}\theta \\\le & {} \Vert Z^0\Vert _{C} + \int _{\tau ^f(\gamma )}^t 2 M a e^{2Ma \,\theta } \Vert Z^0\Vert _{C} \mathrm{\,d}\theta . \end{aligned}$$

Then

$$\begin{aligned} | \mathcal{F}_{[D]}(Z)(\gamma )|\le & {} 2 \Vert Z^0\Vert _{C} \Big (1 + 2 M a \int _0^t e^{2Ma \,\theta } \mathrm{\,d}\theta \Big ) \\= & {} 2 \Vert Z^0\Vert _{C} e^{2Ma \,t}. \end{aligned}$$

Thus $\mathcal{F}_{[D]}(Z)$ fulfills the growth condition in the definition of $\mathcal{K}_{T,a}$.

For any $Z \in \mathcal{K}_{T,a}$, $\gamma _1 = (t_1,\omega _1), \gamma _2 = (t_2,\omega _2) \in \Gamma ^T$ we have

$$\begin{aligned} |\mathcal{F}^S_{[D]}(Z)(\gamma _1) - \mathcal{F}^S_{[D]}(Z)(\gamma _2)|\le & {} | {\bar{S}}^0(\gamma ^f(\gamma _1)) - {\bar{S}}^0(\gamma ^f(\gamma _2))|\\{} & {} + \, \Big |\int _{\tau ^f(\gamma _1)}^{t_1} F^S(\theta ,\omega ^f[\gamma _1](\theta ), D(\theta ), z_1(\theta )) \mathrm{\,d}\theta \\{} & {} - \int _{\tau ^f(\gamma _2)}^{t_2} F^S(\theta ,\omega ^f[\gamma _2](\theta ), D(\theta ), z_2(\theta )) \mathrm{\,d}\theta \Big |, \end{aligned}$$

where $z_i(\theta ):= S(\theta ,\omega ^f[\gamma _i](\theta ))$. Denote $[\tau ', \tau '']:= [\tau ^f(\gamma _1), t_1] \cap [\tau ^f(\gamma _2), t_2]$. Then we split the above integrals into three parts (in each of the integrals only two parts may be non-degenerate). The integration in the first part is on an interval of length $|\tau ^f(\gamma _1) - \tau ^f(\gamma _2)| \le \lambda _\gamma |\gamma _1-\gamma _2|$, and in the third part – of length $|t_1 - t_2| \le |\gamma _1-\gamma _2|$. In view of (6.4) and the growth condition in the definition of $\mathcal{K}_{T,a}$, the integrands can be mojorated by $M a ( 2e^{2Ma} \Vert Z^0\Vert _{C(0,1)})$. Then the sum of these integrals is at most $4Ma (1+\lambda _\gamma ) e^{2 Ma} \Vert Z^0\Vert _{C(0,1)}) |\gamma _1-\gamma _2|$. The integral on $[\tau ', \tau '']$ can be estimated by

$$\begin{aligned}{} & {} \int _{\tau '}^{\tau ''} |F^S(\theta ,\omega ^f[\gamma _1](\theta ), D(\theta ), z_1(\theta )) - F^S(\theta ,\omega ^f[\gamma _2](\theta ), D(\theta ), z_2(\theta ))| \mathrm{\,d}\theta \\{} & {} \le \int _{\tau '}^{\tau ''} L a \Big ( \big (2e^{2M a} \Vert Z^0\Vert _{C(0,1)}\big ) \, |\omega ^f[\gamma _1](\theta ) - \omega ^f[\gamma _2](\theta )| + |z_1(\theta ) - z_2(\theta )| \Big ) \mathrm{\,d}\theta \\{} & {} \le T L \lambda _\omega a \Big ( 2 e^{2M a} \Vert Z^0\Vert _{C(0,1)} + (\Vert Z^0\Vert _{C(0,1)} + \textrm{Lip}(Z^0)) \lambda \Big ) |\gamma _1 - \gamma _2|, \end{aligned}$$

where we make use of (6.4), the growth condition and the Lipschitz property in the definition of $\mathcal{K}_{T,a}$. Combining the obtained estimations and using the same estimations for $\mathcal{F}^{I}_{[D]}(Z)$, we obtain that

$$\begin{aligned}{} & {} |\mathcal{F}_{[D]}(Z)(\gamma _1) - \mathcal{F}_{[D]}(Z)(\gamma _2)| \le \Big (2 \lambda _\gamma \textrm{Lip}(Z^0) + \,8Ma (1+\lambda _\gamma ) e^{2M a}\Vert Z^0\Vert _{C(0,1)} \\{} & {} \quad + \; 2TL \lambda _\omega a \big ( 2 e^{2M a} \Vert Z^0\Vert _{C(0,1)} + (\Vert Z^0\Vert _{C(0,1)} + \textrm{Lip}(Z^0)) \lambda \Big ) |\gamma _1-\gamma _2|\\{} & {} \! = \Big ( (2 \lambda _\gamma + 2TL\lambda _\omega a \, \lambda ) \textrm{Lip}(Z^0) + \big ( 4a e^{2M a} (2 M (1+\lambda _\gamma ) + L\lambda _\omega ) \\{} & {} \quad + 2TL\lambda _\omega a \lambda \big ) \Vert Z^0\Vert _{C(0,1)} \Big ) |\gamma _1-\gamma _2| \\{} & {} \le \Big [\Big ( \frac{1}{2} + \frac{1}{2}\Big ) \lambda \,\textrm{Lip}(Z^0) + \Big ( \frac{1}{2} + \frac{1}{2}\Big ) \lambda \,\Vert Z^0\Vert _{C(0,1)} \Big ] |\gamma _1-\gamma _2| \nonumber \\{} & {} \le \lambda \,(\textrm{Lip}(Z^0) + \Vert Z^0\Vert _{C(0,1)}) |\gamma _1-\gamma _2|, \end{aligned}$$

where in the last inequality we have used (6.7) and (6.8). This completes the proof of the invariance of $\mathcal{K}_{T,a}$.

**4.** (Contractivity of $\mathcal{F}_{[D]}$.) For $Z, Z_1 \in \mathcal{K}_{T,a}$ we have, using (6.5) and (6.7),

$$\begin{aligned} | \mathcal{F}^S_{[D]}(Z)(\gamma ) - \mathcal{F}^S_{[D]}(Z_1)(\gamma ) |\le & {} \int _{\tau ^f(\gamma )}^t L a | Z(s, \omega ^f[\gamma ](s)) - Z_1(s, \omega ^f[\gamma ](s)) | \mathrm{\,d}s \\\le & {} T L a \Vert Z-Z_1\Vert _{C(\Gamma ^T)} \le \frac{1}{2} \Vert Z-Z_1\Vert _{C(\Gamma ^T)}. \end{aligned}$$

According to the Banach contraction mapping theorem, for any function $D \in L^\infty (0,T)$ with $\Vert D \Vert _{L^\infty } + 1 \le a$, there exists a unique $(S,I) = (S[D], I[D]) \in K_{T,D}$ that solves the system (3.7)–(3.8).

**5.** (Properties of (*S*[*D*], *I*[*D*]).) So far we have proved that there exists $T > 0$ such that for any $D \in L^\infty (0,1)$ with $\Vert D\Vert _{L^\infty } + 1 \le a$, the system (3.7)–(3.8) has a Lipschitz continuous solution $Z[D] = (S[D],I[D])$ on $\Gamma ^T$. Here *T* is independent of $Z^0$. The Lipschitz constant of *Z*[*D*] can be estimated by the constant $\lambda ^*:= (\Vert Z^0\Vert _{C(0,1)} + \textrm{Lip}(Z^0)) \lambda \le 2 \textrm{Lip}(Z^0) \lambda$ (see (6.8) and (6.9)).

For a fixed *D* as above, we shorten the notation $Z[D] = (S[D],I[D])$ to $Z = (S,I)$. For any $\gamma = (\tau ,\xi ) \in \Gamma _f$ the function $z^S[\gamma ](t):= S(t, \omega ^f[\gamma ](t))$ satisfies (due to the identities $\omega ^f[t,\omega ^f[\gamma ](t)](s) = \omega ^f[\gamma ](s)$ and $\gamma ^f(\omega ^f[\gamma ](t)) = \gamma$) the relation

$$\begin{aligned} z^S[\gamma ](t) = \int _\tau ^t F^S(s, \omega ^f[\gamma ](s),D(s), z^S[\gamma ](s), I(s,\omega ^f[\gamma ](s))) \mathrm{\,d}s + {\bar{S}}^0(\gamma ), \end{aligned}$$

and an analogical equation is satisfied by $z^I[\gamma '](t):= I(t, \omega ^g[\gamma '](t))$, $\gamma ' \in \Gamma _g$. Differentiating these relations, we obtain the following ODEs satisfied by the Lipschitz functions $z^S[\gamma ]$ and $z^{I}[\gamma ']$ on [0, *T*]:

6.12 $$\begin{aligned} \dot{z}^S[\gamma ](t)= & {} F^S(t, \omega ^f[\gamma ](t),D(t), z^S[\gamma ](t), I(t,\omega ^f[\gamma ](t))), \quad \gamma \in \Gamma _f, \qquad \end{aligned}$$

6.13 $$\begin{aligned} \dot{z}^I[\gamma '](t)= & {} F^I(t, \omega ^g[\gamma '](t),D(t), S(t,\omega ^g[\gamma '](t)), z^I[\gamma '](t)), \quad \gamma ' \in \Gamma _g\qquad \end{aligned}$$

(the so-called equations representing the solution along the characteristic lines). One can inversely express $S(t,\omega ) = z^S[\gamma ^f(t,\omega )](t)$ and similarly for *I*.

Now we shall prove that if *D* is non-negative, then (*S*, *I*) is also non-negative, making use of the property (6.6). Denote

$$\begin{aligned} p(t):= \min \left\{ 0, \min _{\omega \in [0,1]} S(t,\omega ) \right\} , \qquad q(t):= \min \left\{ 0, \min _{\omega \in [0,1]} I(t,\omega ) \right\} . \end{aligned}$$

We have $p(0) = q(0) = 0$ and both functions are Lipschitz continuous. Let $[0,t_p]$ be a maximal sub-interval of [0, *T*] such that $p(t) = 0$ for all $t \in [0,t_p]$. Similarly, let $[0,t_q]$ be the maximal interval on which $q(t) =0$. If $t_p < t_q \le T$, then for every $\gamma \in \Gamma _f$ and $t \in [0,t_q]$ we have $I(t,\omega ^f[\gamma ](t)) \ge 0$, hence $F^S(t,\omega ^f[\gamma ],0,I(t,\omega ^f[\gamma ](t))) \ge 0$. Then, by a standard argument, the set $s \ge 0$ is invariant with respect to (6.12) on $[0,t_q]$ for any $\gamma \in \Gamma _f$, thus $z^S[\gamma ](t) \ge 0$ on $[0, t_q]$. This contradicts the definition of $t_p$ and implies $t_q \le t_p$. Similarly we can prove that $t_p \le t_q$, thus $t_p = t_q =: {\bar{t}}$.

Assume that ${\bar{t}} <T$ and take an arbitrary $\gamma \in \Gamma _f$ and $t \in ({\bar{t}}, T]$. Consider two cases:

(i) $$z^S[\gamma ](t) \ge 0$$;

(ii) $$z^S[\gamma ](t) < 0$$.

In the second case there is a minimal number $t' \in [0,t)$ such that $z^S[\gamma ](s) < 0$ on $(t', t]$. Since $Z^S[\gamma ]({\bar{t}}) \ge p({\hat{t}}) = 0$, we have that $t' \in [{\bar{t}}, t)$ and $z^S[\gamma ](t') = 0$. In the expressions below we skip the first three arguments of $F^S$, namely, $s, \omega ^f[\gamma ](s), D(s)$, since they stay the same in all formulas. We have

$$\begin{aligned} z^S[\gamma ](t)= & {} \int _{t'}^t F^S(z^S[\gamma ](s), I(s,\omega ^f[\gamma ](s))) \mathrm{\,d}s \\= & {} \int _{t'}^t \big (F^S(z^S[\gamma ](s), I(s,\omega ^f[\gamma ](s))) - F^S(0, I(s,\omega ^f[\gamma ](s)))\big ) \mathrm{\,d}s\\{} & {} + \int _{t'}^t F^S(0, I(s,\omega ^f[\gamma ](s))) \mathrm{\,d}s \\\ge & {} -\int _{t'}^t La | z^S[\gamma ](s)| \mathrm{\,d}s - \int _{Q} Ma |I(s,\omega ^f[\gamma ](s))| \mathrm{\,d}s, \end{aligned}$$

where $Q:= \{s \in [t',t]: \, F^S(s, 0, I(s,\omega ^f[\gamma ](s))) < 0\}$. Notice that according to (6.6), $I(s,\omega ^f[\gamma ](s)) < 0$ on this set, hence $-|I(s,\omega ^f[\gamma ](s))| = I(s,\omega ^f[\gamma ](s)) \ge q(t)$. Also $-| z^S[\gamma ](s)| = z^S[\gamma ](s) \ge p(s)$. Then

$$\begin{aligned}{} & {} z^S[\gamma ](t) \ge La \int _{t'}^t p(s) \mathrm{\,d}s + Ma \int _Q q(s) \mathrm{\,d}s \ge C \int _{t'}^t (p(s) + q(s)) \mathrm{\,d}s \\{} & {} \quad \ge C \int _{{\bar{t}}}^t (p(s) + q(s)) \mathrm{\,d}s, \end{aligned}$$

where $C:= a\max \{L,M\}$. Combining the two cases we obtain that

$$\begin{aligned} z^S[\gamma ](t) \ge \min \Big \{ 0, \, C \int _{{\bar{t}}}^t (p(s) + q(s)) \mathrm{\,d}s \Big \} = C \int _{{\bar{t}}}^t (p(s) + q(s)) \mathrm{\,d}s, \end{aligned}$$

Since $\gamma \in \Gamma _f$ is arbitrary, this inequality implies

6.14 $$\begin{aligned} p(t) \ge C \int _{\bar{t}}^\theta (p(s) + q(s)) \mathrm{\,d}s. \end{aligned}$$

By the same argument, a similar inequality is fulfilled by *q*. Summing the two inequalities we obtain that

$$\begin{aligned} p(t) + q(t) \ge 2 C \int _{{\bar{t}}}^t (p(s) + q(s)) \mathrm{\,d}s, \quad t \in [{\bar{t}}, T]. \end{aligned}$$

Since *p* and *q* are Lipschitz continuous non-positive functions, we conclude that $p(t) + q(t) = 0$ on $[{\bar{t}},T]$, hence also on [0, *T*]. Then (6.14) implies that $p(t) = 0$ and similarly $q(t) = 0$. This proves the nonnegativity of *S* and *I*.

The next step is to proof that the solution (*S*[*D*], *I*[*D*]) of (3.7)–(3.8) on [0, *T*] depends in a Lipschitz way on *D* in a sense that will become clear in the next lines. For any two functions $D_1,D_2 \in L^\infty (0,1)$ with $\Vert D_1\Vert _{L^\infty }\,, \, \Vert D_2\Vert _{L^\infty } \le a - 1$, denote $\Delta (t):= \sup _{\omega \in [0,1]} | Z[D_1](t,\omega ) - Z[D_2] (t,\omega )|$. For any $\gamma = (t,\omega ) \in \Gamma ^T$ we have from (6.5) that

$$\begin{aligned} |S[D_1](\gamma ) - S[D_2](\gamma )|\le & {} La \int _{\tau ^f(\gamma )}^t \Big (A (s) \,|D_1(s)- D_2(s)| \\{} & {} \quad + \big |Z[D_1](s,\omega ^f[\gamma ](s))- Z[D_2](s,\omega ^f[\gamma ](s)) \big | \Big ) \mathrm{\,d}s, \end{aligned}$$

where $A(s):= |Z[D_1](s,\omega ^f[\gamma ](s))| \le 2 e^{2\,M aT} \Vert Z^0\Vert _C$, according to the growth condition in the definition of $\mathcal{K}_{T,a}$. A similar inequality holds for *I*. Summing the two, and taking the supremum in $\omega \in [0,1]$ on the left-hand side, we obtain that

$$\begin{aligned} \Delta (t) \le 2 L a\int _{\tau ^f(\gamma )}^t \Big (2 e^{2M aT} \Vert Z^0\Vert _C \;|D_1(s)- D_2(s)| + \Delta (s) \Big ) \mathrm{\,d}s. \end{aligned}$$

Using the Grünwal inequality we obtain that

$$\begin{aligned} \Delta (t) \le \int _{\tau ^f(\gamma )}^t 4La \,e^{2aL(t-s)} e^{2MaT} \Vert Z^0\Vert _C \,|D_1(s)- D_2(s)| \mathrm{\,d}s \le L_Z \Vert D_1 - D_2 \Vert _{L^1(0,t)}, \end{aligned}$$

where $L_Z:= 4L a e^{2aL + 2aM} \Vert Z^0\Vert _C$. This inequality gives the meaning of the Lipschitz property of *Z*[*D*].

**6.** (Proof of the existence claim in Theorem 6.1.) Define the set

$$\begin{aligned} \mathcal{N}_T:= \{ D \in L^1(0,T): \, 0 \le D(t) \le a - 1, \, t \in [0,T] \}. \end{aligned}$$

Then the solution *Z*[*D*] of (3.7)–(3.8), defined in point 4 of the proof, exists for every $D \in \mathcal{N}_T$ and we may define

$$\begin{aligned} \mathcal{G}(D) = \mathcal{D}(Z[D]), \quad D \in \mathcal{N}_T. \end{aligned}$$

Due to the properties of $\mathcal{D}$ and $\mathcal{N}_T$ the latter is invariant with respect $\mathcal{G}$. Apparently, it is a complete metric space. We shall show that the mapping $\mathcal{G}$ is contractive with respect to the norm $\Vert D \Vert _N:= \int _0^T e^{-t N} | D(t) | \mathrm{\,d}t$, where $N > 2 L_D L_Z$. Indeed,

$$\begin{aligned} \Vert \mathcal{G}(D_1) - \mathcal{G}(D_2) \Vert _N= & {} \int _0^T e^{-t N} | \mathcal{D}(Z[D_1])(t) - \mathcal{D}(Z[D_2])(t)| \mathrm{\,d}t \\\le & {} L_D \int _0^T e^{-t N} \max _{\omega \in [0,1]}| Z[D_1](t,\omega ) - Z[D_2](t,\omega )| \mathrm{\,d}t \\\le & {} L_\mathcal{D}L_Z \int _0^T e^{-t N} \Vert D_1 - D_2\Vert _{L^1(0,t)} \mathrm{\,d}t \\\le & {} L_\mathcal{D}L_Z \int _0^T | D_1(s) - D_2(s)| \int _s^T e^{-t N} \mathrm{\,d}t \mathrm{\,d}s \\\le & {} \frac{L_D L_Z}{N} \int _0^T | D_1(s) - D_2(s)| e^{-s N} = \frac{L_D L_Z}{N} \Vert D_1 - D_2\Vert _N \\\le & {} \frac{1}{2} \Vert D_1 - D_2\Vert _N. \end{aligned}$$

Thus $\mathcal{G}$ is a contraction on $\mathcal{N}_T$ hence it has a fix point $D^*$. Obviously the pair $(Z^*:= Z[D^*], D^*)$ satisfies the system (3.7), (3.8), (6.1). Moreover, $Z^* \in \mathcal{K}_{T,a}$ is a Lipschitz function, which completes the proof of the existence part of Theorem 6.1.

**7.** (Proof of the uniqueness claim in Theorem 6.1.) Let for some $T^* > 0$ system (3.7), (3.8), (6.1) has two nonnegative Lipschitz continuous solutions $Z_i = (S_i,I_i), \; i=1,2,$ on $\Gamma ^{T^*}$, satisfying $\mathcal{D}(Z_i) \le a-1$. Without any restriction we may assume that $Z_1$ and $Z_2$ differ from each other for some arbitrary small $t >0$ (otherwise we may compare these solutions starting at a later time chosen so that the solutions immediately decline from each other).

Consider any $T \in (0,T^*]$ (to be fixed later). Denote $D_i:= \mathcal{D}(Z_i)$. Then $|D_i(t)| \le a-1$, $t \in [0,T]$, and using (6.3) we have

$$\begin{aligned} \Vert D_1 - D_2 \Vert _{C(0,T)} \le L_\mathcal{D}\, \Vert Z_1 - Z_2\Vert _{C(\Gamma ^T)}. \end{aligned}$$

Moreover, using (3.7) and (6.5) we have that for any $\gamma = (t,\omega ) \in \Gamma ^T$

$$\begin{aligned} |S_1(\gamma ) - S_2(\gamma )|\le & {} \int _{\tau ^f(\gamma )}^t L a \big ( |Z_1(s,\omega ^f[\gamma ](s))| \, |D_1(s) - D_2(s)|\\{} & {} + |Z_1(s,\omega ^f[\gamma ](s)) - |Z_2(s,\omega ^f[\gamma ](s)) |\, \big ) \mathrm{\,d}s \\\le & {} \int _{\tau ^f(\gamma )}^t L a \big ( \Vert Z_1 \Vert _{C(\Gamma ^T)} \, L_\mathcal{D}\, \Vert Z_1 - Z_2\Vert _{C(\Gamma ^T)} + \Vert Z_1 - Z_2\Vert _{C(\Gamma ^T)} \, \big ) \mathrm{\,d}s \\\le & {} L a T ( L_\mathcal{D}\Vert Z_1 \Vert _{C(\Gamma ^{T^*})} + 1) \,\Vert Z_1 - Z_2\Vert _{C(\Gamma ^T)}. \end{aligned}$$

Let us take the supremum in $\gamma \in \Gamma ^T$ in the right-hand side and then fix *T* so small that $L a T ( L_\mathcal{D}\Vert Z_1 \Vert _{C(\Gamma ^{T^*})} + 1) < 1/2$. We obtain that $\Vert Z_1 - Z_2\Vert _{C(\Gamma ^T)} \le \Vert Z_1 - Z_2\Vert _{C(\Gamma ^T)}/2$, which leads to the contradiction $\Vert Z_1 - Z_2\Vert _{C(\Gamma ^T)} = 0$. The proof of the theorem is complete. $\square$

### Proof of of Theorem 3.1

Now, we consider the specific system (2.3)–(2.7). The conditions (6.4)–(6.6) are apparently fulfilled in this case. Inspecting the proof of Theorem 6.1 we see that the conditions (6.2)–(6.3) are not used in parts 1–5 (they are only used in parts 6 and 7). We have proved (in parts 1–5) that for any $D \in L^\infty (0,T)$ with $0 \le D(t) \le a - 1$ for a.e. $t \in [0,T]$, there exists a non-negative Lipschitz continuous solution $Z[D] \in \mathcal{K}_{T,a}$ of (3.7)–(3.8), hence of (2.4)–(2.7). The existence of *Z*[*D*] was obtained due to the contractivity of the operator $\mathcal{F}_{[D]}$ on $\mathcal{K}_{T,a}$. Hence, *Z*[*D*] can be considered as the uniform limit of the sequence of functions $\{Z_k\}$ generated by

$$\begin{aligned} Z_{k+1} = \mathcal{F}_{[D]}(Z_k), \quad Z_0 = {{\mathcal {Z}}}^\#. \end{aligned}$$

Notice that due to (6.4) and the definition of $\mathcal{F}_{[D]}$ we have

$$\begin{aligned} \Vert Z_{k+1} \Vert _C \le TMa \Vert Z_{k} \Vert _C + \Vert Z^0 \Vert _C, \end{aligned}$$

which implies the estimate

6.15 $$\begin{aligned} \Vert Z_{k} \Vert _C \le 2 \Vert Z^0 \Vert _C, \quad \text{ provided } \text{ that } \, T \le \frac{1}{2 M a}. \end{aligned}$$

Further, we shall choose *T* satisfying the last inequality, in addition to (6.7).

Denote by $\Omega _0$ the set of points in (0, 1) on which $S^0$ or $I^0$ is non-differentiable, together with the points $\omega = 0$ and $\omega = 1$. Denote

$$\begin{aligned} \Gamma ^\#{} & {} := \{ \gamma \in \Gamma ^T: \, \xi ^f(\gamma ) \in \Omega _0 \text{ or } \xi ^g(\gamma ) \in \Omega _0 \} = \{ \omega ^f[(\xi ,0)](t), \, \omega ^g[(\xi ,0)](t): \,\\{} & {} \quad \xi \in \Omega _0, \, t \in [0,T] \}. \end{aligned}$$

This set consists of finite number of curves in $\Gamma ^T$. Observe that the assumption $f', g' < 0$ on (0, 1) implies that the set $\bar{\Gamma }^T = \Gamma ^T \setminus \Gamma ^\#$ consists of finite number of open sets, further called facets. We remind that $\gamma ^f(\gamma )$ and $\gamma ^g(\gamma )$ have Lipschitz derivatives in a neighborhood of $\Gamma ^T$. Then the function $Z_0(\gamma ) = Z^\#(\gamma ) = ({\bar{S}}^0(\gamma ^f(\gamma )), {\bar{I}}^0(\gamma ^g(\gamma )))$ is differentiable with a Lipschitz derivative on each facet of $\bar{\Gamma }^T \setminus \Gamma ^\#$. In addition, for every $\gamma \in \Gamma ^T$ the functions $s \mapsto \sigma (\omega ^f[\gamma ](s))$ and $s \mapsto \sigma (\omega ^g[\gamma ](s))$ are differentiable and have Lipschitz derivatives on every of the finite number of intervals for *s* in which the argument of $\sigma$ belongs to one facet. The same applies to the functions $\rho$ and $\mu$. Thanks to the properties mentioned in this paragraph, we can differentiate $Z_{k+1}$ with respect to $\gamma \in \bar{\Gamma }^T$ using (6.10)–(6.11). Skipping the cumbersome details, we obtain the following relations:

$$\begin{aligned} \textrm{Lip}^\#\Big (\frac{\partial Z_{k+1}}{\partial \gamma }\Big ) \le c_1 + c_2 \textrm{Lip}^\#\Big (\frac{\mathrm{\,d}S^0}{\mathrm{\,d}\omega }\Big ) + Tc \, \textrm{Lip}^\#\Big (\frac{\partial Z_{k}}{\partial \gamma }\Big ), \end{aligned}$$

where $\textrm{Lip}^\#(Q)$ is a common Lipschitz constant of a function *Q* on each facet of $\bar{\Gamma }^T$ (for $Q: \bar{\Gamma }^T \rightarrow \mathbb {R}^2$ which is Lipschitz on every facet), $\textrm{Lip}^\#\Big (\frac{\mathrm{\,d}S^0}{\mathrm{\,d}\omega }\Big )$ is the Lipschitz constant of $\frac{\mathrm{\,d}S^0}{\mathrm{\,d}\omega }$ on each of the intervals of its existence, $c_1$ and $c_2$ are constants (which may depend on $\textrm{Lip}(D)$ and $\Vert Z^0 \Vert _C$), *c* is a constant which is independent of $Z^0$ and *D* with $0\le D \le a-1$. The derivation of this recurrent inequality also uses the fact that $\mathcal{F}_{[D]}$ is an affine mapping of *Z*. Since $\textrm{Lip}^\#\Big (\frac{\mathrm{\,d}S^0}{\mathrm{\,d}\omega }\Big )$ is finite, we obtain inductively that for every *k*

$$\begin{aligned} \textrm{Lip}^\#\Big (\frac{\partial Z_{k+1}}{\partial \gamma }\Big ) \le \Big (c_1 + c_2 \textrm{Lip}^\#\Big (\frac{\mathrm{\,d}S^0}{\mathrm{\,d}\omega }\Big )\Big ) \sum _{j=0}^k (T c)^j \le 2 \Big (c_1 + c_2 \textrm{Lip}^\#\Big (\frac{\mathrm{\,d}S^0}{\mathrm{\,d}\omega }\Big )\Big ), \end{aligned}$$

provided that $2cT \le 1$. We add the last condition to (6.15) and (6.7). The choice of *T* is still independent of the initial distribution $Z^0$ and the particular *D*. For the limit *Z*[*D*] of $Z_k$ we obtain

$$\begin{aligned} \textrm{Lip}^\#\Big (\frac{\partial Z[D]}{\partial \gamma }\Big ) \le 2 \Big (c_1 + c_2 \textrm{Lip}^\#\Big (\frac{\mathrm{\,d}S^0}{\mathrm{\,d}\omega }\Big )\Big ). \end{aligned}$$

Since every horizontal and every vertical line intersects $\Gamma ^\#$ only finite number of times, the partial derivatives of *Z*[*D*] in each of the variables $(t,\omega )$ exist, except a finite number of points, for every value of the other variable.

Using the obtained differentiability properties of the solution (*S*[*D*], *I*[*D*]), we may employ (3.11) to estimate

$$\begin{aligned} \frac{\mathrm{\,d}}{\mathrm{\,d}t} \int _0^1 (S[D](t,\omega ) + I(t,\omega ))\mathrm{\,d}\omega= & {} - \int _0^1 \mu (\omega ) I[D](t,\omega ) \mathrm{\,d}\omega \\\ge & {} - \Vert \mu \Vert _{L^\infty (0,1)} \int _0^1 I[D](t,\omega )\mathrm{\,d}\omega , \end{aligned}$$

hence

$$\begin{aligned} \int _0^1 (S[D](t,\omega ) + I[D](t,\omega ))\mathrm{\,d}\omega\ge & {} 1 - \Vert \mu \Vert _{L^\infty (0,1)} \int _0^t \int _0^1 I[D](t,\omega )\mathrm{\,d}\omega \mathrm{\,d}t \\\ge & {} 1 - T \Vert \mu \Vert _{L^\infty (0,1)} \ge \frac{1}{2}, \end{aligned}$$

where, if necessary, we choose the number *T* even smaller, so that $T \Vert \mu \Vert _{L^\infty (0,1)} \le 1/2$ (still being independent of the distribution of the initial data). The inequality $\int _0^1 I[D](t,\omega )\mathrm{\,d}\omega \le 1$ used above, follows from the obtained decrease of $\int _0^1 (S[D](t,\omega ) + I[D](t,\omega ))\mathrm{\,d}\omega$ starting from value 1 at $t = 0$. So we obtain that

6.16 $$\begin{aligned} \int _0^1 (c_I I[D]((t,\omega ) + c S[D](t,\omega ))\mathrm{\,d}\omega \ge \frac{1}{2} \min \{c_I, c\} > 0, \quad t \in [0,T]. \qquad \end{aligned}$$

Now we return to conditions (6.2)–(6.3). The first one is apparently fulfilled for the mapping $\mathcal{D}$ defined by (2.3) with $a = \max _{\omega \in [0,1]} \iota (\omega )$ (with the convention that $\mathcal{D}(0) = 0$). Condition (6.3) is not fully used in the proof of Theorem 6.1 (part 6). What is used, is the inequality

$$\begin{aligned}{} & {} | \mathcal{D}(Z[D_1])(t) - \mathcal{D}(Z[D_2])(t)| \le L_D \max _{\omega \in [0,1]}| Z[D_1](t,\omega ) - Z[D_2](t,\omega )|, \\{} & {} \quad D_1, \, D_2 \in \mathcal{N}_T. \end{aligned}$$

Due to (6.16) (which holds for every $D \in \mathcal{N}_T$) and the specific form of $\mathcal{D}$, a constant $L_D$ does exist such that the last inequality is fulfilled. For the same reason, $\mathcal{D}(Z[D])(\cdot )$ is Lipschitz continuous for every $D \in \mathcal{N}_T$, uniformly in *D*. Thus the fixed point *D* of $\mathcal{G}$ (which defines a solution *Z*[*D*] of (2.3)–(2.7) in point 6 of the proof) is also Lipschitz continuous. Hence, *Z*[*D*] has the desired differentiability property.

It remains to prove that the solution (*S*, *I*, *D*) can be extended to $[0,\infty )$. We have proved that it exists on [0, *T*] and that *T* is independent of particular distribution of $S^0$ and $I^0$, given that $\int _0^1 (S^0(\omega ) + I^0(\omega )) \mathrm{\,d}\omega = 1$. Taking new initial data ${\tilde{S}}^0(\omega ) = S(T,\omega )/\beta$, ${\tilde{I}}^0(\omega ) = I(T,\omega )/\beta$ with $\beta = \int _0^1 (S(T,\omega ) + I(T,\omega )) \mathrm{\,d}\omega$ (so that the new initial data are normalized), we may apply the obtained existence result: a solution $({\tilde{S}}, {\tilde{I}}, {\tilde{D}})$ exists on [0, *T*]. Observe that the system (2.3)–(2.5) is homogeneous of first order. Then $(S(T+t,\omega ),I(T+t,\omega ),D(T+t)):=( \beta {\tilde{S}}(t,\omega ),\beta {\tilde{I}}(t,\omega ), \beta {\tilde{D}}(t))$ is a continuation of the solution on [0, 2*T*]. The process can be infinitely continued. This proves the existence part of Theorem 3.1.

The uniqueness follows from that in Theorem 6.1 and the existence of a number *a* such that conditions (6.2)–(6.3) are fulfilled. $\square$

## Footnotes

1 A quadratic function *g* is used in this paper, however depending on the current in-body severeness of the infection; in our model the severeness itself depends on the immunity which also influences the time of recovery and hence, the immunity level at.

2 We thank an anonymous referee for pointing out this connection.

3 The assumption can be relaxed by replacing “finite number” with “countable number”.
